# Medicaid Policy Change and Immediate Postpartum Long-Acting Reversible Contraception

**DOI:** 10.1001/jamahealthforum.2024.1359

**Published:** 2024-06-07

**Authors:** Maria I. Rodriguez, Thomas H. A. Meath, Kelsey Watson, Ashley Daly, K. John McConnell, Hyunjee Kim

**Affiliations:** 1 Center for Reproductive Health Equity, Obstetrics & Gynecology, Oregon Health & Science University, Portland; 2 Center for Health Systems Effectiveness, Emergency Medicine, Oregon Health & Science University, Portland

## Abstract

**Importance:**

Improving access to the choice of postpartum contraceptive methods is a national public health priority, and the need is particularly acute within the Medicaid population. One strategy to ensure individuals have access to the full range of contraceptive methods is the provision of a method prior to hospital discharge following a birth episode. Beginning in 2016, some states changed their Medicaid billing policy, allowing separate reimbursement for intrauterine devices and contraceptive implants to increase the provision of long-acting reversible contraceptive (LARC) methods immediately postpartum (IPP).

**Objective:**

To assess the association of a change in Medicaid billing policy with use of IPP LARC.

**Design, Setting, and Participants:**

The cohort study of postpartum Medicaid recipients in 9 treatment and 6 comparison states was conducted from January 2016 to October 2019. Data were analyzed from August 2023 to January 2024.

**Main Outcomes and Measures:**

The primary outcome was use of IPP LARC.

**Results:**

The final sample included 1 378 885 delivery encounters for 1 197 287 Medicaid enrollees occurring in 15 states. Mean age of beneficiaries at delivery was 27 years. The IPP LARC billing policy was associated with a mean increase of 0.74 percentage points (95% CI, 0.30-1.18 percentage points) in the immediate receipt of IPP LARC, with a prepolicy baseline rate of 0.54%. The IPP LARC billing policy was also associated with an overall increase of 1.48 percentage points (95% CI, 0.43-2.73 percentage points) in LARC use by 60 days post partum.

**Conclusions and Relevance:**

In this cohort study, changing Medicaid billing policy to allow for separate reimbursement of LARC devices from the global fee was associated with increased use of IPP LARC, suggesting that this may be a strategy to improve access to the full range of postpartum contraceptive methods.

## Key Points

**Question:**

Was a change in Medicaid policy allowing for reimbursement of long-acting, reversible contraception (LARC) separate from the obstetrics global fee associated with an increased use of LARC immediately postpartum?

**Findings:**

In this cohort study of 1 378 885 deliveries to 1 197 287 Medicaid recipients in 15 states, the change in the Medicaid billing policy was associated with increased use of immediate postpartum LARC.

**Meaning:**

These findings suggest that when the Medicaid billing policy allows for separate reimbursement of LARC, Medicaid recipients may be more likely to receive LARC prior to hospital discharge from childbirth.

## Teaser

This cohort study assesses the association between a change in Medicaid policy allowing for reimbursement of long-acting, reversible contraception (LARC) separate from the obstetrics global fee and use of LARC immediately postpartum.

## Introduction

Ensuring access to a full and informed choice of all contraceptive methods in the postpartum period is fundamental to reproductive autonomy and health.^1,2^ Postpartum contraception is associated with decreased likelihood of maternal and infant morbidity by allowing an individual to choose whether and when to become pregnant again.^3,4^ Both unintended pregnancy and short interpregnancy intervals (pregnancies conceived within 18 months of a prior birth) are associated with adverse maternal health.^4,5,6^

Strategies to improve access to effective contraceptives in the postpartum period are particularly important within the US Medicaid population. Medicaid recipients are at increased risk for unintended pregnancy, short interpregnancy interval births, and severe maternal morbidity.^6,7,8^ Risks for these outcomes can be mitigated by postpartum contraception. However, postpartum contraceptive use within the Medicaid population falls below national goals.^9^ One strategy to ensure individuals have access to the full range of contraceptive methods is provision of contraception prior to hospital discharge after a delivery.

For many years, it has been possible for Medicaid beneficiaries to receive permanent contraception and prescriptions for short-acting methods post partum prior to hospital discharge. However, insurance billing procedures restricted access to long-acting, reversible contraception (LARC) immediately postpartum (IPP), including intrauterine devices (IUDs) or contraceptive implants provided during a hospital admission for childbirth. Traditionally, Medicaid reimbursed for all obstetric care bundled under a global fee; reimbursement for the LARC device outside the obstetric bundle was not feasible.^10,11^ Hospitals were unwilling to provide IPP LARC within the global fee because Medicaid reimbursement for obstetric care is commonly less than the actual costs of providing care,^10^ and the cost for an IUD or implant is high.^12^ However, beginning in 2012, state policymakers began amending Medicaid billing policy to allow the costs of the device to be billed separately from the obstetric global fee.^11,13^ Over the subsequent decade, the majority of states followed suit, with 43 states amending Medicaid policy.

Limited data exist on the effects of these policies on postpartum contraceptive use among Medicaid recipients.^14,15,16,17^ A cross-sectional study from 5 states (Georgia, Iowa, Maryland, New York, and Rhode Island) suggested that the policies allowing separate billing were associated with increased IPP LARC use across all payers at the time of hospital discharge.^17^ Data from 1 state (South Carolina) indicated that reimbursement policies for IPP LARC were associated with increased IPP LARC use, particularly among people with medically complex pregnancies and younger people.^15,18,19^ However, these previous studies used analytic approaches that were unable to account for confounding factors coinciding with the policy change and included only a limited number of states that implemented separate billing policies for IPP LARC.

The objective of our study was to assess the association nationally between changes in Medicaid policy allowing for separate reimbursement for IPP LARC with receipt of LARC within 7 days of delivery (IPP LARC) and by 60 days postpartum. We leveraged a national database of Medicaid claims and used a quasi-experimental design to adjust for temporal changes and potential confounders.

## Methods

### Data Source

This cohort study used a dataset consisting of Medicaid pharmacy, inpatient, and other services claims and enrollment records from release 2 (2016-2018) and release 1 (2019) of the Transformed Medicaid Statistical Information Systems Analytic File (TAF). We followed the Strengthening the Reporting of Observational Studies in Epidemiology (STROBE) reporting guideline. The institutional review board at the Oregon Health & Science University approved this study with a waiver of informed consent because of minimal risk to participants.

### Study Setting

Our study setting included states that implemented IPP LARC separate billing between January 2017 and October 2019 (treatment group), and states that had not implemented the policy as of October 2019 (control group). We identified implementation dates for the policy through a policy review of documents from the Medicaid and Children's Health Insurance Program Payment and Access Commission inventory of state-level Medicaid policies, programs, and initiatives to improve maternity care and outcomes^13^; a bulletin from the Department of Health and Human Services^20^; a website on Medicaid reimbursement for postpartum LARC hosted by the American College of Obstetricians and Gynecologists^21^; and a set of surveys conducted by the Kaiser Family Foundation.^22,23^ Full details on how we assessed policy implementation dates are provided in the eMethods 1 in Supplement 1 and the eTable in Supplement 2.

We first excluded 22 states that implemented IPP LARC separate billing prior to January 2017, as they had less than 12 months of prepolicy period data. We excluded 4 additional states and territories with policies that did not fall into our treatment and control group definitions.^13^ Ten additional states were excluded due to high-concern or unusable TAF databased on the Centers for Medicare & Medicaid Services Data Quality Atlas indicating incomplete or unreliable enrollment records, procedure codes, or diagnosis codes.^24^ Full details on the selection of states in the sample are provided in the eMethods, eTables 1 to 3, and eFigure 1 in Supplement 1.

Among the remaining 15 states, 9 states implemented IPP LARC separate billing across 5 waves: (1) January 2017 (Oregon, Wisconsin, West Virginia, and Virginia), (2) July 2017 (Ohio), (3) January 2018 (New Hampshire), (4) July 2018 (New Jersey), and (5) October 2018 (Michigan and North Carolina). By the end of 2019, 6 states (Colorado, Kansas, Minnesota, Nebraska, North Dakota, and Wyoming) had not implemented the policy and served as our control group for this study.

### Study Cohort Selection

We included delivery encounters for postpartum Medicaid enrollees aged 18 to 44 years living in the 9 treatment states and 6 control states. We excluded deliveries for beneficiaries with restricted benefits due to citizenship status. We also excluded delivery encounters that included diagnosis or procedure codes indicating miscarriage, abortion, ectopic pregnancy, or stillbirth.

### Outcome Measures

Our main outcome was the receipt of IPP LARC, defined as a binary indicator for any inpatient LARC insertions within 7 days of delivery. We identified IPP LARC using the Office of Population Affairs (Department of Health and Human Services) Contraceptive Provision Measure to identify live birth deliveries and any use of IUD or contraceptive implant insertion using procedural and device codes following a delivery.^25^

Our secondary outcomes were (1) receipt of LARC within 60 days, defined as a binary indicator for any outpatient or immediate inpatient LARC insertions within 60 days of delivery; (2) receipt of immediate postpartum permanent contraception, defined as a binary indicator for any permanent contraception method (female sterilization) within 7 days of delivery; and (3) receipt of immediate most or moderately effective contraception, defined as a binary indicator of any most or moderately effective method (female sterilization, implant, IUD, injectable, oral pill, patch, ring, or diaphragm) within 7 days of delivery. All outcomes were identified using diagnosis, procedure, or National Drug Codes from Office of Population Affairs Contraceptive Provision Measure lookup tables for 2016 through 2019. These secondary outcomes allowed us to assess whether changes observed in the primary outcome represented either changes in contraception timing or substitution of contraception method rather than increases in access to people who would not otherwise receive contraceptives.

### Explanatory Variables

We adjusted for the enrollee’s age at the end of each year. We also accounted for patient medical complexity by including Elixhauser comorbidity indicators based on the presence of diagnosis codes on claims within 7 days of the delivery.^26^ We included only 6 of 30 comorbidities that occurred in more than 1% of the sample during every policy wave and month: depression, diabetes without chronic complications, drug misuse, complicated hypertension, chronic pulmonary disease, and obesity.

We could not include each enrollee’s race and ethnicity due to high missing rates in the race and ethnicity fields of TAF. Thus, we adjusted for state-level measures of the proportion of Medicaid-paid deliveries attributable to people of each race and ethnicity group in each year, collected from the Centers for Disease Control and Prevention WONDER dataset.^27^

### Statistical Analysis

Data were analyzed from August 2023 to January 2024. We assessed the association of IPP LARC separate billing policy (hereafter, *policy*) with each of our outcomes using a staggered difference-in-differences model as described in Sun and Abraham.^28^ The model allowed us to account for the varying timing of policy implementation among the treatment groups and accounted for differences in the policy response across time and across implementation waves (eMethods 3 in Supplement 1). We used a linear probability model with a series of interaction terms between binary indicators of each policy implementation wave (waves 1-5) and binary variables for each month relative to the policy implementation (up to 33 months prior to policy implementation and up to 33 months following policy implementation) as primary explanatory variables. The period 3 months prior to the implementation served as the reference point to account for any anticipatory changes prior to implementation. The model also included wave fixed effects to account for time invariant differences between states that implemented IPP LARC separate billing at different times and month fixed effects to account for secular time trends.

After estimating the model, we calculated the average policy effect estimate. We averaged the coefficients of the interaction terms, weighted by the relative sample size in each wave and month.^28^ Additionally, we calculated the average policy effect estimate for each month to assess how it changed over time after its implementation and the average policy effect estimate for each policy implementation wave to assess potential heterogeneity in the policy effect estimates across waves. We clustered SEs at the state level to account for the correlation of observations within states. Further details on the model specifications are provided in eMethods 5 in Supplement 1.

We tested parallel prepolicy time trends for each outcome and policy implementation wave (eMethods 3-5 in Supplement 1). To account for differences in prepolicy time trends between waves, we detrended our outcome variable in each policy implementation wave using the estimated prepolicy time trends from the parallel test model following methods described in prior studies.^29,30,31^ We also ran a sensitivity analysis modeling raw outcomes to compare with the detrended model results and assess the impact of parallel trends (eFigures 5-18 in Supplement 1). All analyses and data management were conducted using R, version 4.2.3 (R Project for Statistical Computing). Two-sided *P* < .05 was considered to be statistically significant.

## Results

**Figure 1.**
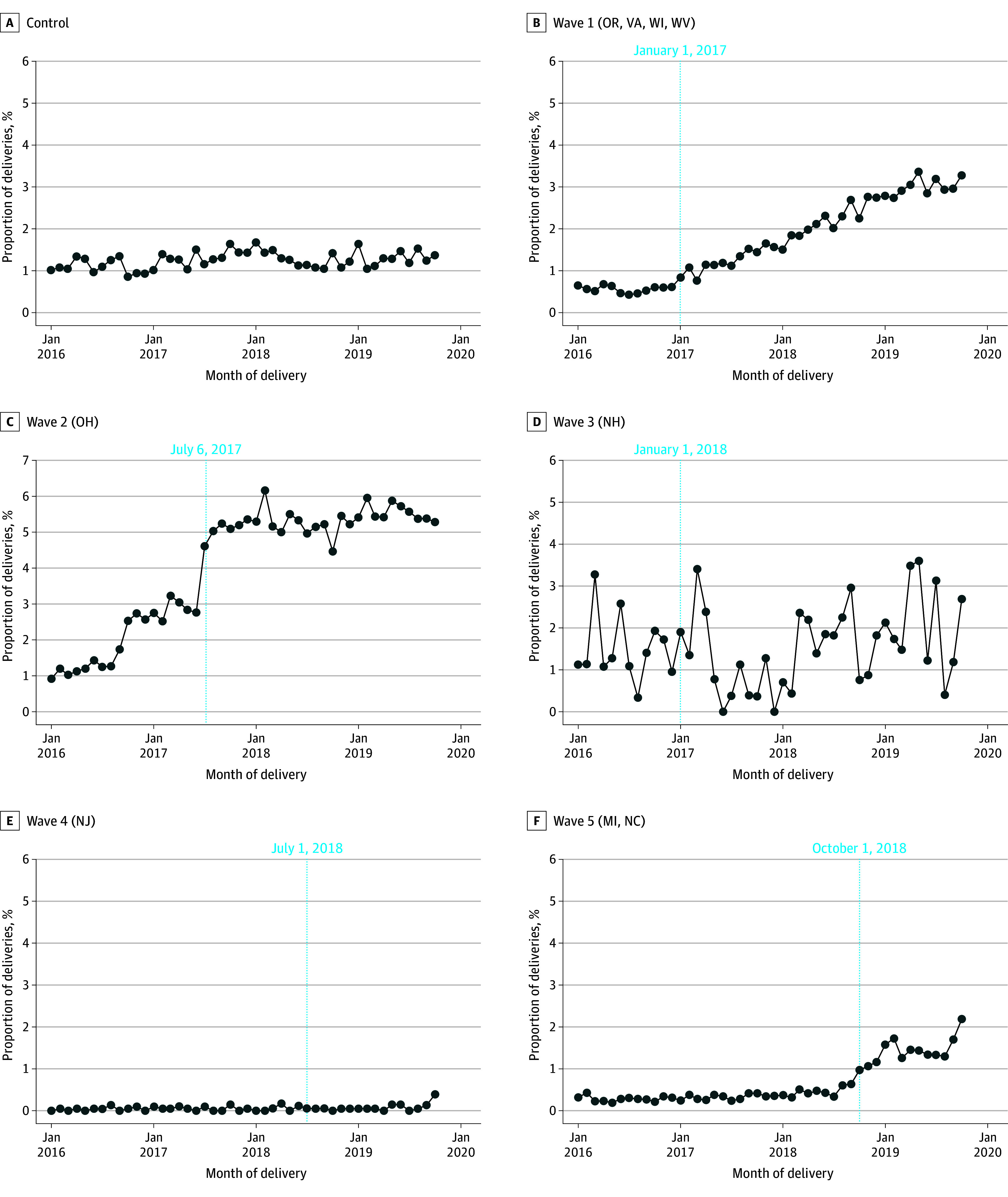
Unadjusted Monthly Rates of Immediate Postpartum Long-Acting Reversible Contraception Over Time Stratified by Policy Implementation Wave Dashed vertical lines represent the month of policy implementation.

The final sample included 1 378 885 delivery encounters for 1 197 287 Medicaid enrollees occurring in 15 states (Table 1). Mean age of beneficiaries at delivery was 27 years. People who delivered in the 9 states that implemented the IPP LARC policy tended to have higher rates of obesity, chronic pulmonary disease, and hypertension compared with those in the control states (Table 1). Unadjusted rates for IPP LARC showed relatively stable trends prior to the policy in most implementation waves and increasing trends in most waves following implementation (Figure 1). The baseline rate in 60-day LARC use among the study population was 10.44%, increasing to 11.72% after the policy change (Table 2). Unadjusted rates for secondary outcomes are presented in eFigures 2-4 in Supplement 1.

**Table 1.** Delivery-Level Demographics of Medicaid Beneficiaries Delivering in 15 States With and Without Separate Medicaid Reimbursement for Immediate Postpartum LARC, 2016-2019^a^

Characteristic	Deliveries, No. (%) (N = 1 378 885)		
	Control (n = 308 538)	Treatment	
		Prepolicy (n = 537 704)	Postpolicy (n = 532 643)
Age of beneficiary at delivery, mean (SD), y	27.64 (5.58)	27.10 (5.44)	27.34 (5.52)
Postpartum LARC use			
Immediate (within 7 d of delivery)	3841 (1.2)	3507 (.7)	14401 (2.7)
Interval (within 60 d of delivery)	42 376 (13.7)	52 110 (9.7)	62 554 (11.7)
Sterilization within 7 d of delivery	20 068 (6.5)	38 955 (7.2)	37 013 (6.9)
Most or moderately effective contraception within 7 d of delivery	28 878 (9.4)	56 169 (1.4)	64 881 (12.2)
Elixhauser comorbidity			
Depression	17 102 (5.5)	23 894 (4.4)	29 906 (5.6)
Diabetes without chronic complications	24 915 (8.1)	42 968 (8.0)	44 649 (8.4)
Drug misuse	16 242 (5.3)	37 793 (7.0)	44 708 (8.4)
Hypertension with chronic complications	12 415 (4.0)	29 413 (5.5)	33 301 (6.3)
Chronic pulmonary disease	17 116 (5.5)	34 564 (6.4)	37 986 (7.1)
Obesity	36 763 (11.9)	77 841 (14.5)	83 925 (15.8)

Abbreviation: LARC, long-acting reversible contraception.

^a^ *P* < .001 for all.

**Table 2.** DiD Model Results Among Medicaid Recipients, 2016-2019

Outcome	Deliveries, % (N = 1 378 885)						DiD estimate, percentage points (95% CI)^c^
	Control states			Treatment states			
	Study start^a^	Study end^b^	Raw difference	Study start^a^	Study end^b^	Raw difference	
IPP LARC	1.02	1.37	0.35	0.54	3.05	2.51	0.74 (0.3 to 1.18)
Interval LARC	13.41	14.11	0.70	10.44	11.72	1.28	1.58 (0.43 to 2.73)
Sterilization within 7 d	5.72	5.68	−0.04	6.89	6.45	−0.44	0.59 (−0.03 to 1.22)
Most or moderately effective contraception within 7 d	8.29	9.00	0.70	10.33	11.96	1.62	0.92 (0.13 to 1.71)

Abbreviations: DiD, difference in differences; IPP, immediate postpartum; LARC, long-acting reversible contraception.

^a^ Raw outcome rates for the first month of the study period (January 2016).

^b^ Raw outcome rates for the last month of the study period (October 2019).

^c^ Estimates are from the staggered implementation model, averaged over all implementation waves and postpolicy periods.

**Figure 2.**
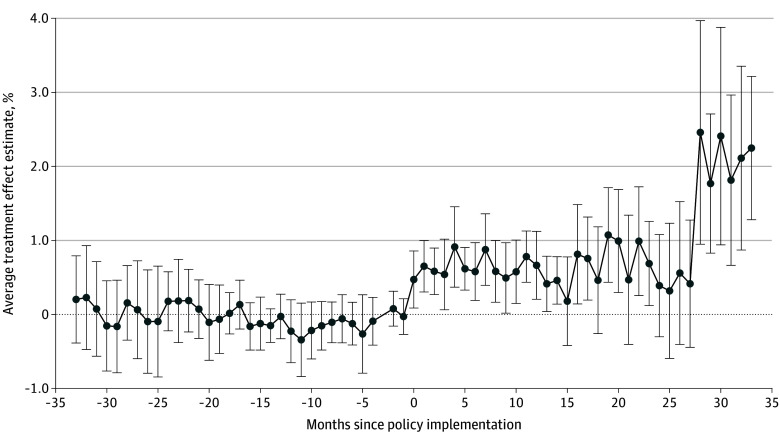
Estimated Changes in Receipt of Immediate Postpartum Long-Acting Reversible Contraception Associated With the Policy for Each Month Before and After Policy Implementation

**Figure 3.**
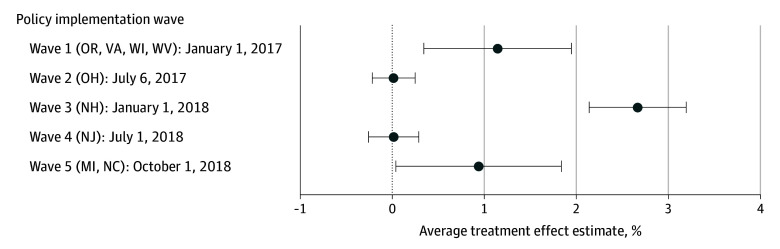
Estimated Changes in Receipt of Immediate Postpartum Long-Acting Reversible Contraception Associated With the Policy for Each Policy Implementation Wave Whiskers represent the 95% CI.

The implementation of the IPP LARC separate billing policy was associated with a mean increase of 0.74 percentage points (95% CI, 0.30-1.18 percentage points) in the receipt of IPP LARC, compared with the prepolicy baseline rate of 0.54% (Table 2). Policy effect estimates for each month suggested that the policy was associated with a gradual increase in rates during the first year following policy implementation (Figure 2). Policy effect estimates stratified by policy implementation wave showed significant associations in only waves 1, 3, and 5, with the largest effect estimates being observed in wave 3 (Figure 3).

Tests of parallel preintervention trends showed evidence of small differences in the prepolicy time trends in waves 2 and 3 (eMethods 5 in Supplement 1). Sensitivity analyses using the raw (vs detrended) outcomes produced qualitatively similar results with marginally larger effect sizes (eMethods 6, 10, and 11 in Supplement 1).

Among the secondary outcomes, implementation of the IPP LARC separate billing policy was associated with a mean increase of 1.58 percentage points (95% CI, 0.43-2.73 percentage points) in the rate of 60-day postpartum LARC from a prepolicy baseline rate of 10.44%. There was no significant difference in the rate of 7-day postpartum sterilization (0.59 percentage points; 95% CI, −0.03 to 1.22 percentage points) from a prepolicy baseline rate of 6.89% and a mean increase of 0.92 percentage points (95% CI, 0.13-1.71 percentage points) in 7-day postpartum most or moderately effective contraception use from a prepolicy baseline rate of 10.33% (Table 2). Month-stratified policy estimates and policy implementation–stratified policy estimates for secondary outcomes are presented in eMethods 7 in Supplement 1.

Evidence of nonparallel trends for each secondary outcome was found in at least 1 wave (eMethods 8 in Supplement 1). Results from sensitivity analyses of the raw outcomes produced qualitatively similar results to those of the detrended models (eMethods 9 in Supplement 1).

## Discussion

In this longitudinal cohort study comparing trends in postpartum LARC use among 9 states that implemented a policy allowing for separate reimbursement for the LARC device from the obstetric global fee, we found an overall 0.74–percentage point increase in IPP LARC use, a relative increase of more than 100% of the prepolicy rate of 0.54%. We observed a gradual increase in policy effect estimates for IPP LARC each month following the policy change through the first year. This was not surprising because changing Medicaid billing policy is just the first step in making IPP LARC a contraceptive choice available for postpartum people. Provision of IPP LARC requires multiple actions throughout the health system that take time to implement.

We examined overall LARC use by 60 days postpartum to assess whether IPP LARC use was potentially reaching people who would not have otherwise used LARC and found the policy to be associated with a 1.58–percentage point increase in overall (60-day) LARC use. Similarly, we found a positive association of the policy change with the broader measure of most or moderately effective contraception within 7 days of delivery, suggesting the observed change was not attributable to a change in contraception type. This was further supported by the null result for 7-day postpartum sterilization, which was the only secondary outcome that did not also include IPP LARC.

Our estimates of the impact of this Medicaid billing policy change align with prior findings in a smaller sample of states providing IPP LARC from 2011 through 2017; changing Medicaid billing policy was associated with a small increase in the provision of IPP LARC.^17^ Our analysis adds to this work by examining results in a larger group of states with more recent data and by including examination of a wider range of contraceptive methods.

Amending billing policies to allow for separate reimbursement of LARC devices outside the global obstetric fee may be a necessary but insufficient step in offering people the choice of IPP LARC. Hospital billing staff need training on how to accurately bill and be reimbursed for the service. Medical and nursing staff need training in patient-centered counseling on IPP LARC provision and insertion technique for IPP IUD placement.^11^ Importantly, devices must be stocked in the inpatient pharmacy and systems developed to order and track the devices.^32^

While national public health goals have focused on reducing the proportion of pregnancies occurring within 18 months of a prior birth through postpartum contraceptive use, there is no target for how many people should be using IPP LARC or any other specific method of contraception postpartum. Nationally, in the Medicaid population, the proportion of people using LARCs by 60 days postpartum is 9.3%, with rates in states ranging from 2.7% to 19.7%.^9^ We observed a similar baseline rate of 10.44% in 60-day LARC use in our study, increasing to 11.72% after the policy change. While IPP LARC accounted for a relatively small proportion of LARC use postpartum, it is a choice that may be valued by people with medical complications or challenges in returning for a postpartum visit. Future research should center on who is using IPP LARC and why.

### Limitations

Our results should be interpreted with the following limitations. First, claims only include data collected for billing purposes; some diagnoses may not have been fully captured. Second, while we verified the policy implementation timing in each state, we did not have information on differences in how states implemented the policy, which may have affected uptake. Third, we did not have information on enrollees’ fertility or contraceptive preferences. Fourth, our study only assessed 4 years of data, which limited our ability to assess longer-term effects of the policy.

## Conclusions

In this cohort study, changing Medicaid billing policy to allow for separate reimbursement of LARC devices from the obstetric global fee was associated with an increase in the use of IPP LARC among Medicaid recipients.

## Notes

Supplement 1. **eMethods 1.** Identification of policy implementation waves **eFigure 1.** Flowchart describing process for identifying policy dates **eMethods 2.** State selection **eTable 1.** Assessment of fully missing eligibility information by state **eTable 2.** OT claims volume data quality assessment for eligible women ages 18 to 44 who gave birth in 2016-2019 **eTable 3.** RX claims volume data quality assessment for eligible women ages 18 to 44 who gave birth in 2016-2019 **eMethods 3.** Staggered Difference-in-differences model specification **eMethods 4.** Unadjusted time trends for secondary outcomes **eFigure 2.** Unadjusted monthly rates of Interval LARC over time stratified by policy implementation waves **eFigure 3.** Unadjusted monthly rates of 7-day postpartum sterilization over time stratified by policy implementation wave **eFigure 4.** Unadjusted monthly rates of 7-day most or moderately effective contraception use over time stratified by policy implementation wave **eMethods 5.** Assessing parallel pre-trends for IPP LARC **eTable 4.** Differences in pre-policy trends of IPP LARC by wave **eMethods 6**. Sensitivity Analysis for IPP LARC models **eFigure 5.** Estimated changes each month pre- and post-policy on use of raw IPP LARC rate **eFigure 6.** Estimated changes associated with the policy for each policy implementation wave on use of raw IPP LARC **eMethods 7.** Month-stratified and wave-stratified results for secondary outcomes **eFigure 7.** Estimated changes each month pre- and post-policy on use of 60-day postpartum LARC **eFigure 8.** Estimated changes associated with the policy for each policy implementation wave on use of 60-day postpartum LARC **eFigure 9.** Estimated changes each month pre- and post-policy on use of 7-day postpartum sterilization **eFigure 10.** Estimated changes associated with the policy for each policy implementation wave on use of 7-day postpartum sterilization **eFigure 11.** Estimated changes each month pre- and post-policy on use of 7-day postpartum most or moderately effective contraception **eFigure 12.** Estimated changes associated with the policy for each policy implementation wave on use of 7-day postpartum most or moderately effective contraception **eMethods 8.** Assessing parallel pre-trends for Secondary Outcomes **eTable 5.** Differences in pre-policy trends of Interval LARC by wave **eTable 6.** Differences in pre-policy trends of 7-day postpartum sterilization by wave **eTable 7.** Differences in pre-policy trends of 7-day postpartum most or moderately effective contraception by wave **eMethods 9.** Sensitivity Analysis for Interval LARC models **eFigure 13.** Estimated changes each month pre- and post-policy on use of raw IPP LARC rate **eFigure 14.** Estimated changes associated with the policy for each policy implementation wave on use of raw IPP LARC **eMethods 10**. Sensitivity Analysis for 7-day postpartum sterilization models **eFigure 15.** Estimated changes each month pre- and post-policy on use of raw IPP LARC rate **eFigure 16.** Estimated changes associated with the policy for each policy implementation wave on use of raw IPP LARC **eMethods 11**. Sensitivity Analysis for 7-day postpartum most or moderately effective contraception models **eFigure 17.** Estimated changes each month pre- and post-policy on use of raw IPP LARC rate **eFigure 18.** Estimated changes associated with the policy for each policy implementation wave on use of raw IPP LARC
eReferences.

Supplement 2.
eTable

Supplement 3. Data Sharing Statement
