# Supplementary material for: Medicaid Policy Change and Immediate Postpartum Long-Acting Reversible Contraception
Source: JAMA Health Forum. 2024 Jun 7;5(6):e241359. doi: 10.1001/jamahealthforum.2024.1359 (PMC11161841; doi:10.1001/jamahealthforum.2024.1359)
Supplement: Supplement 1. — eMethods 1. Identification of policy implementation waves eFigure 1. Flowchart describing process for identifying policy dates eMethods 2. State selection eTable 1. Assessment of fully missing eligibility information by state eTable 2. OT claims volume data quality assessment for eligible women ages 18 to 44 who gave birth in 2016-2019 eTable 3. RX claims volume data quality assessment for eligible women ages 18 to 44 who gave birth in 2016-2019 eMethods 3. Staggered Difference-in-differences model specification eMethods 4. Unadjusted time trends for secondary outcomes eFigure 2. Unadjusted monthly rates of Interval LARC over time stratified by policy implementation waves eFigure 3. Unadjusted monthly rates of 7-day postpartum sterilization over time stratified by policy implementation wave eFigure 4. Unadjusted monthly rates of 7-day most or moderately effective contraception use over time stratified by policy implementation wave eMethods 5. Assessing parallel pre-trends for IPP LARC eTable 4. Differences in pre-policy trends of IPP LARC by wave eMethods 6. Sensitivity Analysis for IPP LARC models eFigure 5. Estimated changes each month pre- and post-policy on use of raw IPP LARC rate eFigure 6. Estimated changes associated with the policy for each policy implementation wave on use of raw IPP LARC eMethods 7. Month-stratified and wave-stratified results for secondary outcomes eFigure 7. Estimated changes each month pre- and post-policy on use of 60-day postpartum LARC eFigure 8. Estimated changes associated with the policy for each policy implementation wave on use of 60-day postpartum LARC eFigure 9. Estimated changes each month pre- and post-policy on use of 7-day postpartum sterilization eFigure 10. Estimated changes associated with the policy for each policy implementation wave on use of 7-day postpartum sterilization eFigure 11. Estimated changes each month pre- and post-policy on use of 7-day postpartum most or moderately effective contraception eFigure 12. E [file jamahealthforum-e241359-s001.pdf]

Supplemental Online Content

Rodriguez MI, Meath TA, Watson K, Daly A, McConnell KJ, Kim H. Medicaid policy change and immediate postpartum long-acting reversible contraception. *JAMA Health Forum.* Published online June 7, 2024. doi:10.1001/jamahealthforum.2024.1359

eMethods 1. Identification of policy implementation waves

eFigure 1. Flowchart describing process for identifying policy dates

eMethods 2. State selection

eTable 1. Assessment of fully missing eligibility information by state

eTable 2. OT claims volume data quality assessment for eligible women ages 18 to 44 who gave birth in 2016-2019

eTable 3. RX claims volume data quality assessment for eligible women ages 18 to 44 who gave birth in 2016-2019

eMethods 3. Staggered Difference-in-differences model specification

eMethods 4. Unadjusted time trends for secondary outcomes

eFigure 2. Unadjusted monthly rates of Interval LARC over time stratified by policy implementation waves

eFigure 3. Unadjusted monthly rates of 7-day postpartum sterilization over time stratified by policy implementation wave

eFigure 4. Unadjusted monthly rates of 7-day most or moderately effective contraception use over time stratified by policy implementation wave

eMethods 5. Assessing parallel pre-trends for IPP LARC

eTable 4. Differences in pre-policy trends of IPP LARC by wave

eMethods 6. Sensitivity Analysis for IPP LARC models

eFigure 5. Estimated changes each month pre- and post-policy on use of raw IPP LARC rate

eFigure 6. Estimated changes associated with the policy for each policy implementation wave on use of raw IPP LARC

eMethods 7. Month-stratified and wave-stratified results for secondary outcomes

eFigure 7. Estimated changes each month pre- and post-policy on use of 60-day postpartum LARC

© 2024 Rodriguez MI et al. *JAMA Health Forum.*

eFigure 8. Estimated changes associated with the policy for each policy implementation wave on use of 60-day postpartum LARC

eFigure 9. Estimated changes each month pre- and post-policy on use of 7-day postpartum sterilization

eFigure 10. Estimated changes associated with the policy for each policy implementation wave on use of 7-day postpartum sterilization

eFigure 11. Estimated changes each month pre- and post-policy on use of 7-day postpartum most or moderately effective contraception

eFigure 12. Estimated changes associated with the policy for each policy implementation wave on use of 7-day postpartum most or moderately effective contraception

eMethods 8. Assessing parallel pre-trends for Secondary Outcomes

eTable 5. Differences in pre-policy trends of Interval LARC by wave

eTable 6. Differences in pre-policy trends of 7-day postpartum sterilization by wave

eTable 7. Differences in pre-policy trends of 7-day postpartum most or moderately effective contraception by wave

eMethods 9. Sensitivity Analysis for Interval LARC models

eFigure 13. Estimated changes each month pre- and post-policy on use of raw IPP LARC rate

eFigure 14. Estimated changes associated with the policy for each policy implementation wave on use of raw IPP LARC

eMethods 10. Sensitivity Analysis for 7-day postpartum sterilization models

eFigure 15. Estimated changes each month pre- and post-policy on use of raw IPP LARC rate

eFigure 16. Estimated changes associated with the policy for each policy implementation wave on use of raw IPP LARC

eMethods 11. Sensitivity Analysis for 7-day postpartum most or moderately effective contraception models

eFigure 17. Estimated changes each month pre- and post-policy on use of raw IPP LARC rate

eFigure 18. Estimated changes associated with the policy for each policy implementation wave on use of raw IPP LARC

© 2024 Rodriguez MI et al. *JAMA Health Forum.*

eReferences.

This supplemental material has been provided by the authors to give readers additional information about their work.

© 2024 Rodriguez MI et al. *JAMA Health Forum.*

Supplement Section 1. Identification of policy implementation waves

A team of policy analysts performed a review of state policies to identify start dates for state Medicaid policies that allowed for separate reimbursement for immediate postpartum long-acting reversible contraception (LARC) in hospital settings. Only states with sufficient data quality as measured using DQ Atlas assessments (Supplement Section 2) were reviewed. The steps used to identify policies and start dates is described below and in Supplement Figure 1. Full details of the policy review results for each state is provided in Supplement Table 1.

1.**For each state, we checked for LARC device reimbursement policies on three websites**. We compared responses for consistency.

A. **MACPAC** – (3/2020) Mathematica, under contract, compiled a spreadsheet of Medicaid Initiatives in states including LARC policies with start dates and original language[1].
B. **DHHS CMS Informational Bulletin** (4/8/16) - a state agency policy scan of innovative state LARC reimbursement strategies[2].
C. **ACOG Website** – (updated 4/2022) -compiled links to state agency Medicaid LARC policies[3].

2. **If no specific policy information was available, we performed an Internet search for policies** using various search terms combinations including “State” “Medicaid,” “LARC,” “Hospital,” “Inpatient,” “Reimbursement,” “IUD,” and “Device.”

3. **We prioritized using original source text from state Medicaid agency policy guidelines, rules and bulletins on LARC reimbursement to determine policy start date and policy details**. The next priority level was using information from secondary sources, such as policy scans from MACPAC, a research publication, or other policy summary. In two instances, ID and NV, we contacted state Medicaid agencies to get clarity on policy information.

4. **To validate our findings, we compared KFF surveys on State Medicaid Coverage for LARC Reimbursement for 2015[4] and 2021[5].** These surveys indicated whether LARC device reimbursement was separate or global (under the maternity global DRG) for both hospital and provider reimbursement. Although survey data usually corresponded with our findings, there were several instances when the responses contradicted with original state Medicaid agency policy guidance. KFF seemed generally accurate, but not completely reliable. Other limitations of KFF data were checkbox responses only (separate/global) and no indication of policy start dates.

5. **We noted, under “Summary,” state agency policy start dates based on original/secondary state agency policy documentation, prioritized finding hospital reimbursement policies, and noted when KFF had contradictory or additional info., not found in our policy scan.** For example, we indicated the policy start date for Oregon was 1/1/17, based on state health authority information of separate hospital reimbursement for postpartum LARC. If we found state agency policy guidance on a separate hospital

© 2024 Rodriguez MI et al. *JAMA Health Forum.*

reimbursement policy, we did not search for a separate provider reimbursement policy. In our summary, we use policy start date of 1/1/17 for hospital reimbursement and noted that provider reimbursement might have started in 2015 or earlier per KFF surveys.

6. **Based on the information found in steps 1-5, the principle investigator assigned each state to one of three groups:** (1) Treatment states – those that implemented a policy during the study period, (2) Control states – states that did not implement the policy or implemented the policy following the end of the study period, (3) Excluded states – those that either implemented the policy prior to the start of the study period or those that could not be classified into the other two groups.

© 2024 Rodriguez MI et al. *JAMA Health Forum.*

Supplement eFigure 1. Flowchart describing process for identifying policy dates

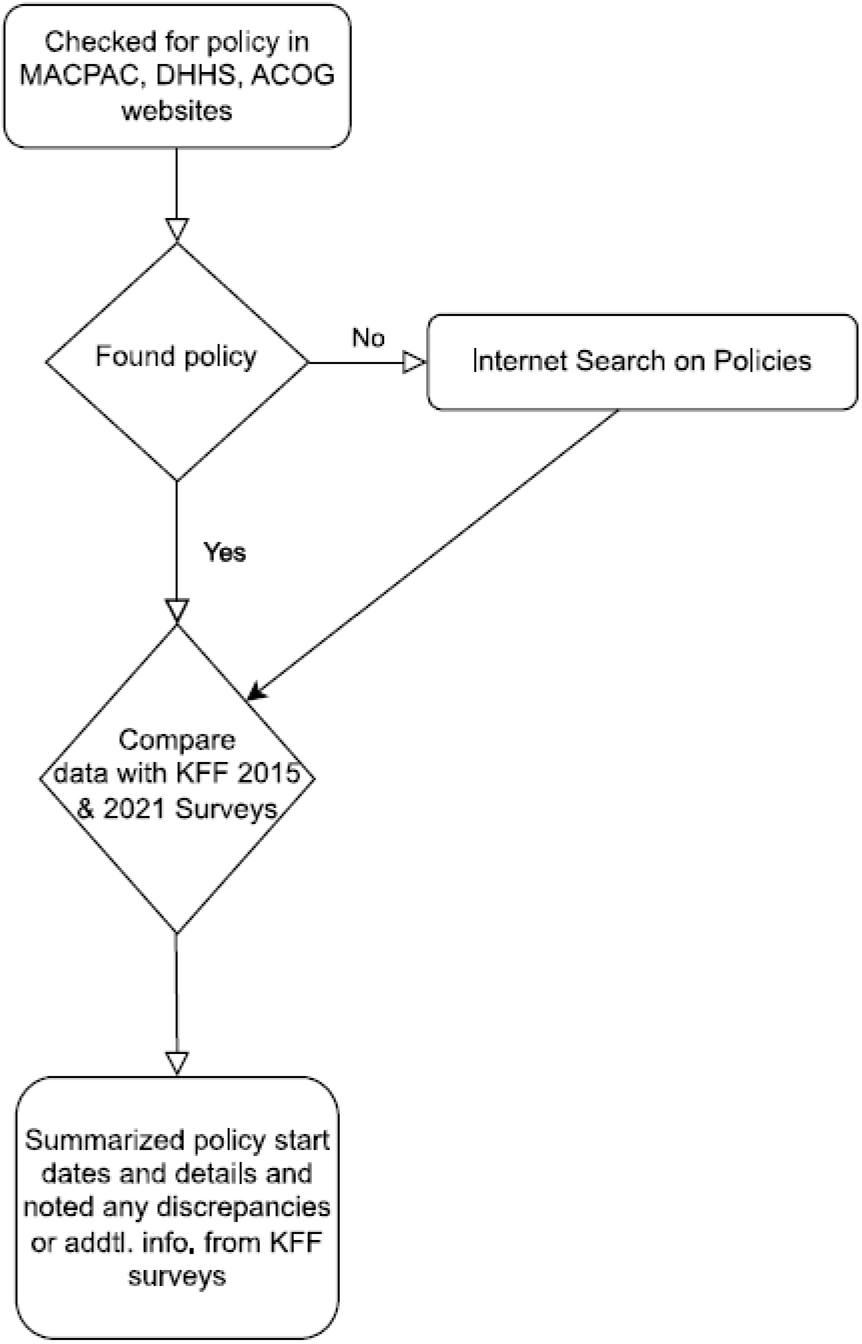

© 2024 Rodriguez MI et al. *JAMA Health Forum.*

Supplement Section 2. State selection

To select states with adequate data quality for analysis we used assessments published by the Centers for Medicare & Medicaid Services DQ Atlas[6] and supplemented our own analyses of the data quality where necessary. We began with all 50 states and the District of Columbia and looked at data quality assessments for TAF RIF Release 2 from 2016, 2017, and 2018 and TAF RIF Release 1 from 2019.

After excluding 22 states that had already implemented an IPP LARC separate billing policy prior to 2016 (Arizona, California, Connecticut, Delaware, Georgia, Hawaii, Idaho, Illinois, Indiana, Iowa, Louisiana, Missouri, Montana, New Mexico, New York, Oklahoma, Pennsylvania, South Carolina, South Dakota, Texas, Vermont, Washington) and 4 states and territories with an unclear policy or policy date (Alaska, District of Columbia, Massachusetts, Nevada), 25 states remained for data quality assessment.

Six states classified as unusable or high concern in any of the relevant assessments for any of the measurement years were excluded from our analysis:

- 2 states with high concern Total Medicaid and CHIP Enrollment (Maine and Rhode Island).
- 2 states with unusable procedure codes on inpatient claims (Kentucky and Maryland).
- 1 state with unusable diagnosis codes on inpatient claims (Tennessee).
- 1 state with unusable procedure codes on professional Other Services claims (Utah).

To assess the remaining 19 states, we conducted two additional assessments of overall TAF data quality. First, we looked at the proportion of enrollment records missing all eligibility information (MISG_ELGBLTY_DATA_IND = 1) and classified states from Low Concern to Unusable based on the same cut-points used in the DQ Atlas assessments of missing gender and missing age on enrollment records (Supplement eTable 2).

Second, we calculated the Claims Volume assessments for our population of interest (Supplement eTable 3 and eTable 4). Because variation in claims volume is not necessarily a sign of poor data quality but may still flag problematic states, we only considered those states classified as unusable on this measure for exclusion.

From these assessments we excluded an additional four states:

- 2 states with high concern (> 20%) missing eligibility records (Alabama and Arkansas)
- 1 state with unusable Other Services (OT) claims volume in our population (Florida)
- 1 state with unusable pharmacy (RX) claims volume in our population (Mississippi)

In total we excluded 35 states and the District of Columbia from analysis based on inadequate data quality or IPP LARC policies. Our remaining cohort included 15 states (Colorado, Kansas, Michigan, Minnesota, Nebraska, New Hampshire, New Jersey, North Carolina, North Dakota, Ohio, Oregon, Virginia, West Virginia, Wisconsin, Wyoming).

© 2024 Rodriguez MI et al. *JAMA Health Forum.*

Supplement eTable 1. Assessment of fully missing eligibility information by state

Year	State	DQ Assessment	# Beneficiaries	% Beneficiaries with Missing Eligibility Record
2016	Alabama	Medium concern	1,505,574	10.5
2016	Alaska	Low concern	209,614	0.1
2016	Arizona	Low concern	2,407,731	1.3
2016	Arkansas	High concern	1,674,720	30.4
2016	California	Low concern	18,314,987	2.2
2016	Colorado	Low concern	1,656,088	0.4
2016	Connecticut	Low concern	1,064,823	0.1
2016	Delaware	Low concern	296,083	0.2
2016	District of Columbia	Low concern	285,341	0.7
2016	Florida	Low concern	5,254,568	1.3
2016	Georgia	Low concern	2,497,727	0.1
2016	Hawaii	Low concern	424,949	0.4
2016	Idaho	Low concern	373,052	2.1
2016	Illinois	Low concern	3,766,853	0.6
2016	Indiana	Low concern	1,865,381	1
2016	Iowa	Low concern	864,440	5.3
2016	Kansas	Low concern	521,091	0.4
2016	Kentucky	Low concern	1,658,555	0.2
2016	Louisiana	Low concern	1,748,158	0
2016	Maine	Low concern	331,147	0
2016	Maryland	Low concern	1,537,892	0.1
2016	Massachusetts	Low concern	2,275,071	1.6
2016	Michigan	Low concern	3,115,922	6.1
2016	Minnesota	Low concern	1,432,163	2.8
2016	Mississippi	Low concern	913,766	0.3
2016	Missouri	Low concern	1,236,569	0.4
2016	Montana	Low concern	284,454	2.7
2016	Nebraska	Low concern	306,932	0.2
2016	Nevada	Low concern	846,124	0.6
2016	New Hampshire	Low concern	260,814	0.1
2016	New Jersey	Low concern	2,234,871	5.2
2016	New Mexico	Low concern	972,603	0.1
2016	New York	Low concern	8,004,690	0.1
2016	North Carolina	Low concern	2,427,017	0.3
2016	North Dakota	Low concern	124,855	0.6
2016	Ohio	Low concern	3,651,749	4.3
2016	Oklahoma	Low concern	1,045,953	2
2016	Oregon	Low concern	1,429,788	0.2

© 2024 Rodriguez MI et al. *JAMA Health Forum.*

2016	Pennsylvania	Low concern	3,457,135	3.8
2016	Rhode Island	Low concern	361,841	0.3
2016	South Carolina	Low concern	1,420,355	0.4
2016	South Dakota	Low concern	155,372	0.1
2016	Tennessee	Low concern	1,823,196	1.5
2016	Texas	Low concern	6,130,716	2.9
2016	Utah	Low concern	449,705	1.8
2016	Vermont	Low concern	227,475	0.2
2016	Virginia	Low concern	1,441,406	0.9
2016	Washington	Low concern	2,209,083	0.3
2016	West Virginia	Low concern	702,053	1.2
2016	Wisconsin	Low concern	1,440,341	1.5
2016	Wyoming	Low concern	106,963	0.1
2017	Alabama	Medium concern	1,583,388	14.7
2017	Alaska	Low concern	233,670	0
2017	Arizona	Low concern	2,350,966	0.5
2017	Arkansas	High concern	1,585,184	24.9
2017	California	Low concern	17,886,931	2.2
2017	Colorado	Low concern	1,700,854	0.3
2017	Connecticut	Low concern	1,086,153	0.2
2017	Delaware	Low concern	291,772	0.1
2017	District of Columbia	Low concern	287,186	0.3
2017	Florida	Low concern	5,325,008	0.7
2017	Georgia	Low concern	2,499,086	0.1
2017	Hawaii	Low concern	422,411	0.9
2017	Idaho	Low concern	383,951	3
2017	Illinois	Low concern	3,682,744	0.1
2017	Indiana	Low concern	1,823,845	0.3
2017	Iowa	Low concern	819,993	0.1
2017	Kansas	Low concern	521,691	0.3
2017	Kentucky	Low concern	1,721,085	0.4
2017	Louisiana	Low concern	1,846,114	0
2017	Maine	Low concern	324,369	0
2017	Maryland	Low concern	1,600,579	0.1
2017	Massachusetts	Low concern	2,272,404	0.1
2017	Michigan	Low concern	2,944,735	0.8
2017	Minnesota	Low concern	1,423,050	4.3
2017	Mississippi	Low concern	899,667	0.3
2017	Missouri	Low concern	1,266,882	0.5
2017	Montana	Low concern	379,496	0.2
2017	Nebraska	Low concern	310,618	0.2
2017	Nevada	Low concern	869,896	0.3

© 2024 Rodriguez MI et al. *JAMA Health Forum.*

2017	New Hampshire	Low concern	262,401	1
2017	New Jersey	Medium concern	2,397,310	11
2017	New Mexico	Low concern	1,010,727	0.1
2017	New York	Low concern	8,102,569	0.1
2017	North Carolina	Low concern	2,522,552	0.5
2017	North Dakota	Low concern	127,354	0.9
2017	Ohio	Low concern	3,671,348	4
2017	Oklahoma	Low concern	1,070,786	4.2
2017	Oregon	Low concern	1,366,394	0.1
2017	Pennsylvania	Low concern	3,621,605	4.6
2017	Rhode Island	Low concern	384,188	0.2
2017	South Carolina	Low concern	1,465,488	0.4
2017	South Dakota	Low concern	153,838	0.1
2017	Tennessee	Medium concern	2,288,850	18.1
2017	Texas	Low concern	6,022,153	1.4
2017	Utah	Low concern	436,958	1.7
2017	Vermont	Low concern	209,021	0.1
2017	Virginia	Low concern	1,445,108	0.6
2017	Washington	Low concern	2,231,975	0.3
2017	West Virginia	Low concern	688,009	0.4
2017	Wisconsin	Low concern	1,402,531	8.6
2017	Wyoming	Low concern	94,073	0.1
2018	Alabama	High concern	1,785,528	24
2018	Alaska	Low concern	246,856	0.1
2018	Arizona	Low concern	2,324,198	0.4
2018	Arkansas	Low concern	1,152,527	0.6
2018	California	Low concern	17,259,966	1.9
2018	Colorado	Low concern	1,666,828	0.6
2018	Connecticut	Low concern	1,097,641	0.2
2018	Delaware	Low concern	294,484	0.1
2018	District of Columbia	Low concern	294,772	0.5
2018	Florida	Low concern	5,239,274	0.3
2018	Georgia	Low concern	2,588,750	0.7
2018	Hawaii	Low concern	418,787	1.1
2018	Idaho	Low concern	367,388	0.1
2018	Illinois	Low concern	3,638,871	0.2
2018	Indiana	Low concern	1,848,347	1.4
2018	Iowa	Low concern	809,174	0.2
2018	Kansas	Low concern	512,851	0.3
2018	Kentucky	Low concern	1,734,002	0.3
2018	Louisiana	Low concern	1,867,965	0
2018	Maine	Low concern	325,405	0

© 2024 Rodriguez MI et al. *JAMA Health Forum.*

2018	Maryland	Low concern	1,623,847	0.2
2018	Massachusetts	Low concern	2,247,777	0.1
2018	Michigan	Low concern	2,951,026	0.6
2018	Minnesota	Low concern	1,430,441	6.1
2018	Mississippi	Low concern	882,116	0.3
2018	Missouri	Low concern	1,248,554	0.4
2018	Montana	Low concern	358,779	0.1
2018	Nebraska	Low concern	312,002	0.1
2018	Nevada	Low concern	878,600	0.3
2018	New Hampshire	Low concern	253,238	0.2
2018	New Jersey	Medium concern	2,499,387	15.3
2018	New Mexico	Low concern	978,618	0
2018	New York	Low concern	8,198,039	0
2018	North Carolina	Low concern	2,593,784	0.4
2018	North Dakota	Low concern	126,386	1
2018	Ohio	Low concern	3,462,339	1.2
2018	Oklahoma	Low concern	1,048,631	4.3
2018	Oregon	Low concern	1,303,604	0.2
2018	Pennsylvania	Low concern	3,658,824	2.4
2018	Rhode Island	Low concern	385,188	2.7
2018	South Carolina	Low concern	1,469,214	0.3
2018	South Dakota	Low concern	151,505	0.1
2018	Tennessee	High concern	2,342,471	21.1
2018	Texas	Low concern	5,930,218	1.1
2018	Utah	Low concern	422,186	0.4
2018	Vermont	Low concern	201,599	0.1
2018	Virginia	Low concern	1,496,868	0.7
2018	Washington	Low concern	2,193,204	0.4
2018	West Virginia	Low concern	673,058	1.2
2018	Wisconsin	Low concern	1,391,491	0.3
2018	Wyoming	Low concern	90,615	0.1
**2019**	Alabama	High concern	1,862,088	27.5
2019	Alaska	Low concern	257,655	0.2
2019	Arizona	Low concern	2,284,084	0.2
2019	Arkansas	Low concern	1,122,655	0.7
2019	California	Low concern	16,636,071	1.4
2019	Colorado	Low concern	1,617,745	0.9
2019	Connecticut	Low concern	1,118,478	0.1
2019	Delaware	Low concern	294,978	0.1
2019	District of Columbia	Low concern	289,532	0.2
2019	Florida	Low concern	5,063,676	0.7
2019	Georgia	Low concern	2,574,731	0.1

© 2024 Rodriguez MI et al. *JAMA Health Forum.*

2019	Hawaii	Low concern	406,649	1.1
2019	Idaho	Low concern	353,386	0.1
2019	Illinois	Low concern	3,485,221	0.3
2019	Indiana	Low concern	1,818,487	0.2
2019	Iowa	Low concern	822,560	0
2019	Kansas	Low concern	505,416	0.2
2019	Kentucky	Low concern	1,698,912	0.3
2019	Louisiana	Low concern	1,892,152	0
2019	Maine	Low concern	355,047	0
2019	Maryland	Low concern	1,610,473	0.1
2019	Massachusetts	Low concern	2,172,070	0.2
2019	Michigan	Low concern	2,948,236	0.6
2019	Minnesota	Low concern	1,382,683	4.3
2019	Mississippi	Low concern	855,885	0.2
2019	Missouri	Low concern	1,194,625	0.3
2019	Montana	Low concern	365,311	0.1
2019	Nebraska	Low concern	310,492	0.2
2019	Nevada	Low concern	867,619	0.2
2019	New Hampshire	Low concern	248,299	0.1
2019	New Jersey	Low concern	2,173,180	3.9
2019	New Mexico	Low concern	952,111	0
2019	New York	Low concern	8,281,504	0.1
2019	North Carolina	Low concern	2,619,089	0.4
2019	North Dakota	Low concern	123,678	0.9
2019	Ohio	Low concern	3,331,426	0.9
2019	Oklahoma	Low concern	1,036,476	4.3
2019	Oregon	Low concern	1,309,103	0.1
2019	Pennsylvania	Low concern	3,667,935	1.5
2019	Rhode Island	High concern	380,347	24.1
2019	South Carolina	Low concern	1,460,877	0.2
2019	South Dakota	Low concern	148,441	0.2
2019	Tennessee	High concern	2,278,950	22.7
2019	Texas	Low concern	5,760,195	0.6
2019	Utah	Low concern	424,428	0.2
2019	Vermont	Low concern	194,696	0.1
2019	Virginia	Low concern	1,779,562	0.9
2019	Washington	Low concern	2,149,654	0.2
2019	West Virginia	Low concern	666,570	2.4
2019	Wisconsin	Low concern	1,375,678	0.3
2019	Wyoming	Low concern	87,941	0.2

© 2024 Rodriguez MI et al. *JAMA Health Forum.*

Supplement eTable 2. OT claims volume data quality assessment for eligible women ages 18 to 44 who gave birth in 2016-2019

State	Data Year	DQ Assessment	Assessment of OT File Header Record Volume	Assessment of OT File Line Record Volume	Assessment of Avg OT Line Records per Header	OT File Header Records per 1,000 Enrolled Months as % of National Median	OT File Line Records per 1,000 Enrolled Months as % of National Median	Avg OT File Line Records per Header Record as % of National Median	OT File Header Records per 1,000 Enrolled Months	National Median: Header Records per 1,000 Enrolled Months in OT File	OT File Line Records per 1,000 Enrolled Months	National Median: Line Records per 1,000 Enrolled Months in OT File	Avg OT File Line Records per Header Record	National Median: Avg Line Records per Header in OT File
Alabama	2016	Medium concern	Medium concern	Medium concern	Low concern	51.1	62.7	114.3	1433.2	2805.0	3197.0	5096.9	2.2	2.0
Alaska	2016	Low concern	Low concern	Low concern	Low concern	108.4	115.8	99.4	3041.7	2805.0	5900.6	5096.9	1.9	2.0
Arizona	2016	Low concern	Low concern	Low concern	Low concern	114.2	89.4	73.0	3202.5	2805.0	4558.8	5096.9	1.4	2.0
Arkansas	2016	High concern	High concern	High concern	Low concern	41.8	48.3	107.6	1173.3	2805.0	2462.3	5096.9	2.1	2.0
California	2016	Low concern	Low concern	Low concern	Low concern	123.5	129.9	98.0	3463.3	2805.0	6620.6	5096.9	1.9	2.0
Colorado	2016	Medium concern	Medium concern	Low concern	Low concern	74.7	80.2	100.0	2094.7	2805.0	4086.3	5096.9	2.0	2.0
Connecticut	2016	Low concern	Low concern	Low concern	Low concern	104.3	133.6	119.3	2924.5	2805.0	6808.6	5096.9	2.3	2.0
Delaware	2016	Medium concern	Low concern	Medium concern	Low concern	136.1	153.7	105.2	3818.6	2805.0	7833.7	5096.9	2.1	2.0
District of Columbia	2016	Low concern	Low concern	Low concern	Low concern	99.3	113.7	106.7	2784.2	2805.0	5795.9	5096.9	2.1	2.0
Florida	2016	Low concern	Low concern	Low concern	Low concern	101.0	110.7	102.1	2832.6	2805.0	5642.1	5096.9	2.0	2.0

© 2024 Rodriguez MI et al. *JAMA Health Forum.*

Georgia	2016	Medium concern	Medium concern	Low concern	Low concern	74.3	77.1	96.7	2083.4	2805.0	3929.7	5096.9	1.9	2.0
Hawaii	2016	Medium concern	Medium concern	Low concern	Low concern	69.2	78.2	105.3	1940.5	2805.0	3987.1	5096.9	2.1	2.0
Idaho	2016	Medium concern	Medium concern	Medium concern	Low concern	74.8	74.3	92.5	2099.4	2805.0	3786.7	5096.9	1.8	2.0
Illinois	2016	Low concern	Low concern	Low concern	Low concern	106.6	98.0	85.6	2990.8	2805.0	4995.9	5096.9	1.7	2.0
Indiana	2016	Low concern	Low concern	Low concern	Low concern	98.6	117.3	110.8	2765.0	2805.0	5977.7	5096.9	2.2	2.0
Iowa	2016	Low concern	Low concern	Low concern	Low concern	85.4	84.4	92.0	2396.1	2805.0	4302.0	5096.9	1.8	2.0
Kansas	2016	Low concern	Low concern	Low concern	Low concern	75.0	80.8	100.3	2104.1	2805.0	4117.1	5096.9	2.0	2.0
Kentucky	2016	Low concern	Low concern	Low concern	Low concern	103.9	108.1	97.0	2913.1	2805.0	5511.1	5096.9	1.9	2.0
Louisiana	2016	High concern	High concern	Low concern	Low concern	201.9	111.7	51.6	5663.1	2805.0	5695.1	5096.9	1.0	2.0
Maine	2016	Medium concern	Medium concern	Medium concern	Low concern	156.8	173.5	103.0	4398.6	2805.0	8841.6	5096.9	2.0	2.0
Maryland	2016	High concern	High concern	Medium concern	Low concern	237.2	155.6	61.1	6653.3	2805.0	7929.6	5096.9	1.2	2.0
Massachusetts	2016	High concern	High concern	Low concern	Low concern	207.8	134.6	60.3	5830.0	2805.0	6859.6	5096.9	1.2	2.0
Michigan	2016	Low concern	Low concern	Low concern	Low concern	100.3	124.5	115.6	2814.4	2805.0	6344.6	5096.9	2.3	2.0
Minnesota	2016	Low concern	Low concern	Low concern	Low concern	107.4	128.4	111.3	3014.0	2805.0	6543.4	5096.9	2.2	2.0
Mississippi	2016	High concern	Low concern	High concern	High concern	80.6	17.4	20.1	2261.1	2805.0	888.8	5096.9	0.4	2.0
Missouri	2016	Low concern	Low concern	Low concern	Low concern	96.1	100.0	96.9	2696.7	2805.0	5096.9	5096.9	1.9	2.0
Montana	2016	Low concern	Low concern	Low concern	Low concern	79.9	90.8	105.9	2240.3	2805.0	4629.0	5096.9	2.1	2.0
Nebraska	2016	Low concern	Low concern	Low concern	Low concern	81.4	89.1	102.0	2283.0	2805.0	4541.9	5096.9	2.0	2.0

© 2024 Rodriguez MI et al. *JAMA Health Forum.*

Nevada	2016	Low concern	Low concern	Low concern	Low concern	77.5	75.1	90.3	2173.6	2805.0	3826.9	5096.9	1.8	2.0
New Hampshire	2016	Low concern	Low concern	Low concern	Low concern	92.7	98.8	99.3	2599.4	2805.0	5035.5	5096.9	1.9	2.0
New Jersey	2016	High concern	High concern	Low concern	Low concern	246.0	135.4	51.3	6901.4	2805.0	6903.8	5096.9	1.0	2.0
New Mexico	2016	Low concern	Low concern	Low concern	Low concern	81.4	103.9	119.0	2282.2	2805.0	5297.6	5096.9	2.3	2.0
New York	2016	Low concern	Low concern	Low concern	Low concern	108.4	136.3	117.1	3039.8	2805.0	6946.5	5096.9	2.3	2.0
North Carolina	2016	Low concern	Low concern	Low concern	Low concern	78.8	103.5	122.4	2210.9	2805.0	5277.4	5096.9	2.4	2.0
North Dakota	2016	Low concern	Low concern	Low concern	Low concern	97.8	98.6	93.9	2743.5	2805.0	5024.6	5096.9	1.8	2.0
Ohio	2016	Medium concern	Low concern	Medium concern	Low concern	121.6	151.3	115.9	3410.6	2805.0	7712.8	5096.9	2.3	2.0
Oklahoma	2016	Medium concern	Medium concern	Low concern	Low concern	72.5	80.8	103.8	2033.7	2805.0	4119.4	5096.9	2.0	2.0
Oregon	2016	Low concern	Low concern	Low concern	Low concern	91.6	97.6	99.2	2570.5	2805.0	4973.0	5096.9	1.9	2.0
Pennsylvania	2016	Low concern	Low concern	Low concern	Low concern	125.1	128.1	95.3	3509.7	2805.0	6527.8	5096.9	1.9	2.0
Rhode Island	2016	Low concern	Low concern	Low concern	Low concern	126.0	90.9	67.2	3533.4	2805.0	4630.6	5096.9	1.3	2.0
South Carolina	2016	Low concern	Low concern	Low concern	Low concern	94.4	105.0	103.5	2649.2	2805.0	5349.8	5096.9	2.0	2.0
South Dakota	2016	Low concern	Low concern	Low concern	Low concern	119.2	89.5	69.9	3344.9	2805.0	4561.1	5096.9	1.4	2.0
Tennessee	2016	Low concern	Low concern	Low concern	Low concern	81.9	87.5	99.5	2296.2	2805.0	4457.7	5096.9	1.9	2.0
Texas	2016	Low concern	Low concern	Low concern	Low concern	104.3	132.4	118.3	2924.7	2805.0	6747.1	5096.9	2.3	2.0
Utah	2016	Medium concern	Medium concern	Medium concern	Low concern	51.5	62.1	112.4	1444.2	2805.0	3166.3	5096.9	2.2	2.0
Vermont	2016	Low concern	Low concern	Low concern	Low concern	106.8	93.6	81.6	2996.0	2805.0	4771.6	5096.9	1.6	2.0

© 2024 Rodriguez MI et al. *JAMA Health Forum.*

Virginia	2016	Low concern	Low concern	Low concern	Low concern	128.1	90.6	65.9	3593.9	2805.0	4619.6	5096.9	1.3	2.0
Washington	2016	Low concern	Low concern	Low concern	Low concern	101.6	111.9	102.6	2850.1	2805.0	5703.7	5096.9	2.0	2.0
West Virginia	2016	Low concern	Low concern	Low concern	Low concern	106.8	136.4	119.0	2994.6	2805.0	6952.9	5096.9	2.3	2.0
Wisconsin	2016	Low concern	Low concern	Low concern	Low concern	100.0	102.6	95.6	2805.0	2805.0	5230.1	5096.9	1.9	2.0
Wyoming	2016	Medium concern	Medium concern	Low concern	Low concern	66.6	78.2	109.4	1867.3	2805.0	3985.9	5096.9	2.1	2.0
Alabama	2017	Medium concern	Medium concern	Medium concern	Low concern	51.2	59.1	111.4	1454.6	2841.1	3229.9	5466.8	2.2	2.0
Alaska	2017	Low concern	Low concern	Low concern	Low concern	97.3	99.3	98.5	2765.6	2841.1	5428.4	5466.8	2.0	2.0
Arizona	2017	Low concern	Low concern	Low concern	Low concern	125.7	99.5	76.5	3570.7	2841.1	5441.3	5466.8	1.5	2.0
Arkansas	2017	High concern	High concern	Medium concern	Low concern	48.2	50.0	100.2	1370.1	2841.1	2734.2	5466.8	2.0	2.0
California	2017	Low concern	Low concern	Low concern	Low concern	130.2	125.7	93.2	3698.9	2841.1	6870.6	5466.8	1.9	2.0
Colorado	2017	Medium concern	Medium concern	Low concern	Low concern	73.4	77.2	101.5	2086.7	2841.1	4219.1	5466.8	2.0	2.0
Connecticut	2017	Low concern	Low concern	Low concern	Low concern	105.5	127.0	116.2	2998.7	2841.1	6944.5	5466.8	2.3	2.0
Delaware	2017	Low concern	Low concern	Low concern	Low concern	130.5	129.5	95.8	3707.4	2841.1	7077.8	5466.8	1.9	2.0
District of Columbia	2017	Low concern	Low concern	Low concern	Low concern	97.2	112.4	111.8	2760.3	2841.1	6145.9	5466.8	2.2	2.0
Florida	2017	High concern	High concern	High concern	Low concern	23.3	25.5	105.8	661.1	2841.1	1393.9	5466.8	2.1	2.0
Georgia	2017	Medium concern	Medium concern	Medium concern	Low concern	67.9	64.8	92.2	1929.2	2841.1	3543.3	5466.8	1.8	2.0
Hawaii	2017	Low concern	Low concern	Low concern	Low concern	77.4	83.2	103.8	2198.9	2841.1	4549.1	5466.8	2.1	2.0
Idaho	2017	Low concern	Low concern	Low concern	Low concern	79.6	84.3	102.3	2260.8	2841.1	4609.7	5466.8	2.0	2.0

© 2024 Rodriguez MI et al. *JAMA Health Forum.*

Illinois	2017	Low concern	Low concern	Low concern	Low concern	125.1	106.3	82.1	3554.1	2841.1	5813.7	5466.8	1.6	2.0
Indiana	2017	Low concern	Low concern	Low concern	Low concern	96.9	102.1	101.8	2751.9	2841.1	5582.3	5466.8	2.0	2.0
Iowa	2017	Low concern	Low concern	Low concern	Low concern	92.5	78.1	81.5	2628.8	2841.1	4268.1	5466.8	1.6	2.0
Kansas	2017	Low concern	Low concern	Low concern	Low concern	80.5	83.2	99.8	2286.0	2841.1	4547.2	5466.8	2.0	2.0
Kentucky	2017	Low concern	Low concern	Low concern	Low concern	100.6	103.3	99.2	2857.2	2841.1	5646.8	5466.8	2.0	2.0
Louisiana	2017	High concern	High concern	Low concern	Low concern	220.0	114.9	50.5	6251.8	2841.1	6284.0	5466.8	1.0	2.0
Maine	2017	Medium concern	Low concern	Medium concern	Low concern	148.2	156.3	101.8	4211.5	2841.1	8542.6	5466.8	2.0	2.0
Maryland	2017	High concern	High concern	Low concern	Low concern	230.0	142.8	60.0	6535.0	2841.1	7808.1	5466.8	1.2	2.0
Massachusetts	2017	High concern	High concern	Low concern	Low concern	213.3	136.6	61.8	6061.1	2841.1	7466.6	5466.8	1.2	2.0
Michigan	2017	Low concern	Low concern	Low concern	Low concern	100.2	118.9	114.6	2845.9	2841.1	6499.9	5466.8	2.3	2.0
Minnesota	2017	Low concern	Low concern	Low concern	Low concern	114.0	125.1	106.0	3240.1	2841.1	6839.6	5466.8	2.1	2.0
Mississippi	2017	Low concern	Low concern	Low concern	Low concern	82.3	99.8	117.1	2337.9	2841.1	5456.6	5466.8	2.3	2.0
Missouri	2017	Low concern	Low concern	Low concern	Low concern	104.4	102.8	95.0	2967.0	2841.1	5618.6	5466.8	1.9	2.0
Montana	2017	Low concern	Low concern	Low concern	Low concern	85.7	88.8	100.0	2435.8	2841.1	4853.0	5466.8	2.0	2.0
Nebraska	2017	Low concern	Low concern	Low concern	Low concern	85.2	91.5	103.7	2420.5	2841.1	5002.4	5466.8	2.1	2.0
Nevada	2017	Medium concern	Medium concern	Medium concern	Low concern	73.4	71.9	94.5	2086.3	2841.1	3929.8	5466.8	1.9	2.0
New Hampshire	2017	Low concern	Low concern	Low concern	Low concern	91.3	94.5	100.0	2594.0	2841.1	5166.5	5466.8	2.0	2.0
New Jersey	2017	High concern	High concern	Low concern	Low concern	259.3	134.8	50.2	7366.7	2841.1	7369.1	5466.8	1.0	2.0

© 2024 Rodriguez MI et al. *JAMA Health Forum.*

New Mexico	2017	Low concern	Low concern	Low concern	Low concern	81.9	100.5	118.5	2326.9	2841.1	5493.8	5466.8	2.4	2.0
New York	2017	Low concern	Low concern	Low concern	Low concern	109.2	121.0	107.0	3102.1	2841.1	6615.4	5466.8	2.1	2.0
North Carolina	2017	Low concern	Low concern	Low concern	Low concern	81.6	96.4	114.1	2318.4	2841.1	5271.3	5466.8	2.3	2.0
North Dakota	2017	Low concern	Low concern	Low concern	Low concern	105.1	102.5	94.1	2987.4	2841.1	5601.2	5466.8	1.9	2.0
Ohio	2017	Medium concern	Low concern	Medium concern	Low concern	127.3	152.9	116.0	3616.3	2841.1	8356.0	5466.8	2.3	2.0
Oklahoma	2017	Medium concern	Medium concern	Low concern	Low concern	74.6	78.5	101.6	2119.5	2841.1	4291.9	5466.8	2.0	2.0
Oregon	2017	Low concern	Low concern	Low concern	Low concern	97.5	97.0	96.1	2770.6	2841.1	5303.8	5466.8	1.9	2.0
Pennsylvania	2017	Low concern	Low concern	Low concern	Low concern	124.0	115.5	90.0	3522.6	2841.1	6316.4	5466.8	1.8	2.0
Rhode Island	2017	Low concern	Low concern	Low concern	Low concern	133.2	84.8	61.5	3783.4	2841.1	4633.8	5466.8	1.2	2.0
South Carolina	2017	Low concern	Low concern	Low concern	Low concern	95.6	100.0	101.1	2715.0	2841.1	5466.8	5466.8	2.0	2.0
South Dakota	2017	Low concern	Low concern	Low concern	Low concern	120.7	85.4	68.4	3428.6	2841.1	4670.1	5466.8	1.4	2.0
Tennessee	2017	Low concern	Low concern	Low concern	Low concern	82.8	85.3	99.5	2351.3	2841.1	4663.5	5466.8	2.0	2.0
Texas	2017	Low concern	Low concern	Low concern	Low concern	106.6	128.1	116.1	3027.4	2841.1	7001.0	5466.8	2.3	2.0
Utah	2017	Low concern	Low concern	Low concern	Low concern	78.5	82.3	101.2	2230.7	2841.1	4496.8	5466.8	2.0	2.0
Vermont	2017	Medium concern	Medium concern	Low concern	Low concern	151.6	111.5	71.0	4306.5	2841.1	6094.4	5466.8	1.4	2.0
Virginia	2017	Low concern	Low concern	Low concern	Low concern	142.1	94.2	64.0	4038.3	2841.1	5151.8	5466.8	1.3	2.0
Washington	2017	Low concern	Low concern	Low concern	Low concern	100.0	106.0	102.4	2841.9	2841.1	5796.3	5466.8	2.0	2.0
West Virginia	2017	Low concern	Low concern	Low concern	Low concern	100.0	119.1	115.0	2841.1	2841.1	6508.5	5466.8	2.3	2.0

© 2024 Rodriguez MI et al. *JAMA Health Forum.*

Wisconsin	2017	Low concern	Low concern	Low concern	Low concern	109.8	104.1	91.6	3118.4	2841.1	5692.7	5466.8	1.8	2.0
Wyoming	2017	Low concern	Low concern	Low concern	Low concern	84.3	95.2	109.0	2395.8	2841.1	5202.4	5466.8	2.2	2.0
Alabama	2018	Medium concern	Medium concern	Medium concern	Low concern	54.5	56.4	100.0	1596.9	2932.8	3213.0	5695.0	2.0	2.0
Alaska	2018	Low concern	Low concern	Low concern	Low concern	105.1	107.7	98.9	3082.6	2932.8	6133.2	5695.0	2.0	2.0
Arizona	2018	Low concern	Low concern	Low concern	Low concern	120.6	95.4	76.4	3537.4	2932.8	5435.1	5695.0	1.5	2.0
Arkansas	2018	Medium concern	Medium concern	Medium concern	Low concern	56.1	57.7	99.3	1643.9	2932.8	3284.4	5695.0	2.0	2.0
California	2018	Low concern	Low concern	Low concern	Low concern	128.3	124.7	93.9	3762.2	2932.8	7104.3	5695.0	1.9	2.0
Colorado	2018	Low concern	Low concern	Low concern	Low concern	75.6	81.2	103.6	2217.7	2932.8	4624.5	5695.0	2.1	2.0
Connecticut	2018	Low concern	Low concern	Low concern	Low concern	104.7	130.3	120.2	3070.4	2932.8	7423.3	5695.0	2.4	2.0
Delaware	2018	Low concern	Low concern	Low concern	Low concern	132.9	130.4	94.7	3897.5	2932.8	7426.2	5695.0	1.9	2.0
District of Columbia	2018	Low concern	Low concern	Low concern	Low concern	104.0	116.8	108.4	3049.7	2932.8	6650.3	5695.0	2.2	2.0
Florida	2018	Unusable	Unusable	Unusable	Low concern	5.7	7.6	127.6	168.5	2932.8	432.5	5695.0	2.6	2.0
Georgia	2018	Medium concern	Medium concern	Medium concern	Low concern	63.7	61.9	93.9	1867.3	2932.8	3526.2	5695.0	1.9	2.0
Hawaii	2018	Low concern	Low concern	Low concern	Low concern	75.0	82.4	106.0	2199.8	2932.8	4690.5	5695.0	2.1	2.0
Idaho	2018	Low concern	Low concern	Low concern	Low concern	79.2	84.1	102.5	2324.0	2932.8	4790.8	5695.0	2.1	2.0
Illinois	2018	Low concern	Low concern	Low concern	Low concern	113.6	100.0	84.9	3332.5	2932.8	5695.0	5695.0	1.7	2.0
Indiana	2018	Low concern	Low concern	Low concern	Low concern	93.8	99.2	102.0	2752.0	2932.8	5649.0	5695.0	2.1	2.0
Iowa	2018	Low concern	Low concern	Low concern	Low concern	92.4	80.5	84.1	2708.5	2932.8	4583.1	5695.0	1.7	2.0

© 2024 Rodriguez MI et al. *JAMA Health Forum.*

Kansas	2018	Low concern	Low concern	Low concern	Low concern	81.2	84.2	100.0	2381.6	2932.8	4793.6	5695.0	2.0	2.0
Kentucky	2018	Low concern	Low concern	Low concern	Low concern	100.0	104.0	100.4	2932.8	2932.8	5925.5	5695.0	2.0	2.0
Louisiana	2018	High concern	High concern	Low concern	High concern	229.2	118.5	49.9	6720.9	2932.8	6750.8	5695.0	1.0	2.0
Maine	2018	Low concern	Low concern	Low concern	Low concern	139.1	146.9	101.9	4080.6	2932.8	8365.1	5695.0	2.0	2.0
Maryland	2018	High concern	High concern	Low concern	Low concern	224.2	136.4	58.7	6573.9	2932.8	7765.2	5695.0	1.2	2.0
Massachusetts	2018	Medium concern	Medium concern	Low concern	Low concern	196.5	129.7	63.7	5762.9	2932.8	7387.2	5695.0	1.3	2.0
Michigan	2018	Low concern	Low concern	Low concern	Low concern	96.5	115.4	115.3	2831.3	2932.8	6570.2	5695.0	2.3	2.0
Minnesota	2018	Low concern	Low concern	Low concern	Low concern	113.4	126.6	107.7	3327.0	2932.8	7208.8	5695.0	2.2	2.0
Mississippi	2018	Low concern	Low concern	Low concern	Low concern	80.7	97.6	116.8	2365.9	2932.8	5559.4	5695.0	2.3	2.0
Missouri	2018	Low concern	Low concern	Low concern	Low concern	108.4	105.8	94.2	3178.0	2932.8	6024.2	5695.0	1.9	2.0
Montana	2018	Low concern	Low concern	Low concern	Low concern	89.0	88.9	96.3	2611.3	2932.8	5060.3	5695.0	1.9	2.0
Nebraska	2018	Low concern	Low concern	Low concern	Low concern	80.9	84.7	101.1	2372.3	2932.8	4825.4	5695.0	2.0	2.0
Nevada	2018	Medium concern	Medium concern	Medium concern	Low concern	70.2	70.9	97.5	2058.2	2932.8	4036.1	5695.0	2.0	2.0
New Hampshire	2018	Low concern	Low concern	Low concern	Low concern	82.4	89.6	104.9	2416.1	2932.8	5101.7	5695.0	2.1	2.0
New Jersey	2018	High concern	High concern	Low concern	High concern	248.6	128.1	49.7	7290.6	2932.8	7293.2	5695.0	1.0	2.0
New Mexico	2018	Low concern	Low concern	Low concern	Low concern	82.5	102.5	119.9	2419.0	2932.8	5836.2	5695.0	2.4	2.0
New York	2018	Low concern	Low concern	Low concern	Low concern	110.0	120.7	105.9	3226.8	2932.8	6874.5	5695.0	2.1	2.0
North Carolina	2018	Low concern	Low concern	Low concern	Low concern	79.4	92.9	112.9	2329.1	2932.8	5292.9	5695.0	2.3	2.0

© 2024 Rodriguez MI et al. *JAMA Health Forum.*

North Dakota	2018	Low concern	Low concern	Low concern	Low concern	105.7	105.5	96.3	3101.3	2932.8	6010.3	5695.0	1.9	2.0
Ohio	2018	Medium concern	Low concern	Medium concern	Low concern	129.3	159.4	119.0	3792.7	2932.8	9079.0	5695.0	2.4	2.0
Oklahoma	2018	Medium concern	Medium concern	Medium concern	Low concern	71.8	74.9	100.7	2105.0	2932.8	4265.4	5695.0	2.0	2.0
Oregon	2018	Low concern	Low concern	Low concern	Low concern	111.0	103.0	89.5	3255.7	2932.8	5865.0	5695.0	1.8	2.0
Pennsylvania	2018	Low concern	Low concern	Low concern	Low concern	122.0	118.1	93.5	3577.5	2932.8	6728.4	5695.0	1.9	2.0
Rhode Island	2018	High concern	High concern	Low concern	Low concern	227.0	124.6	53.0	6657.7	2932.8	7095.0	5695.0	1.1	2.0
South Carolina	2018	Low concern	Low concern	Low concern	Low concern	92.8	95.8	99.6	2720.5	2932.8	5453.4	5695.0	2.0	2.0
South Dakota	2018	Low concern	Low concern	Low concern	Low concern	117.1	81.7	67.3	3434.5	2932.8	4650.0	5695.0	1.4	2.0
Tennessee	2018	Low concern	Low concern	Low concern	Low concern	83.2	84.7	98.3	2439.1	2932.8	4824.7	5695.0	2.0	2.0
Texas	2018	Low concern	Low concern	Low concern	Low concern	106.2	128.0	116.4	3114.0	2932.8	7292.3	5695.0	2.3	2.0
Utah	2018	Medium concern	Medium concern	Low concern	Low concern	73.1	77.3	102.0	2144.6	2932.8	4402.4	5695.0	2.1	2.0
Vermont	2018	Low concern	Low concern	Low concern	Low concern	143.9	105.2	70.6	4220.7	2932.8	5992.1	5695.0	1.4	2.0
Virginia	2018	Low concern	Low concern	Low concern	Low concern	132.0	88.0	64.3	3872.4	2932.8	5013.5	5695.0	1.3	2.0
Washington	2018	Low concern	Low concern	Low concern	Low concern	98.7	102.6	100.3	2895.0	2932.8	5844.3	5695.0	2.0	2.0
West Virginia	2018	Low concern	Low concern	Low concern	Low concern	80.8	92.9	111.0	2369.9	2932.8	5291.7	5695.0	2.2	2.0
Wisconsin	2018	Low concern	Low concern	Low concern	Low concern	111.4	105.1	91.1	3267.5	2932.8	5986.6	5695.0	1.8	2.0
Wyoming	2018	Low concern	Low concern	Low concern	Low concern	83.1	93.9	109.1	2436.3	2932.8	5348.3	5695.0	2.2	2.0
Alabama	2019	High concern	High concern	High concern	Low concern	44.9	46.8	99.2	1391.0	3095.5	2777.4	5937.9	2.0	2.0

© 2024 Rodriguez MI et al. *JAMA Health Forum.*

Alaska	2019	Low concern	Low concern	Low concern	Low concern	98.9	101.2	97.5	3062.2	3095.5	6011.2	5937.9	2.0	2.0
Arizona	2019	Low concern	Low concern	Low concern	Low concern	115.2	93.1	77.0	3564.8	3095.5	5525.3	5937.9	1.5	2.0
Arkansas	2019	Medium concern	Medium concern	Medium concern	Low concern	58.0	60.9	100.0	1796.2	3095.5	3615.3	5937.9	2.0	2.0
California	2019	Low concern	Low concern	Low concern	Low concern	121.0	125.2	98.6	3744.1	3095.5	7433.7	5937.9	2.0	2.0
Colorado	2019	Low concern	Low concern	Low concern	Low concern	76.1	84.0	105.2	2354.4	3095.5	4986.5	5937.9	2.1	2.0
Connecticut	2019	Low concern	Low concern	Low concern	Low concern	102.0	130.3	121.7	3158.9	3095.5	7738.3	5937.9	2.4	2.0
Delaware	2019	Low concern	Low concern	Low concern	Low concern	134.9	139.2	98.3	4176.9	3095.5	8268.2	5937.9	2.0	2.0
District of Columbia	2019	Low concern	Low concern	Low concern	Low concern	107.4	119.2	105.8	3323.7	3095.5	7079.7	5937.9	2.1	2.0
Florida	2019	Low concern	Low concern	Low concern	Low concern	78.2	75.4	91.9	2420.4	3095.5	4476.1	5937.9	1.8	2.0
Georgia	2019	Medium concern	Medium concern	Low concern	Low concern	69.8	77.4	105.8	2159.4	3095.5	4597.5	5937.9	2.1	2.0
Hawaii	2019	Medium concern	Medium concern	Low concern	Low concern	69.9	77.2	105.3	2162.7	3095.5	4583.8	5937.9	2.1	2.0
Idaho	2019	Low concern	Low concern	Low concern	Low concern	76.8	83.5	103.6	2378.0	3095.5	4957.4	5937.9	2.1	2.0
Illinois	2019	Low concern	Low concern	Low concern	Low concern	115.5	102.9	85.0	3573.9	3095.5	6112.8	5937.9	1.7	2.0
Indiana	2019	Medium concern	Medium concern	Low concern	Low concern	72.5	77.8	102.2	2244.5	3095.5	4617.1	5937.9	2.1	2.0
Iowa	2019	Low concern	Low concern	Low concern	Low concern	88.5	81.6	87.8	2740.9	3095.5	4846.5	5937.9	1.8	2.0
Kansas	2019	Low concern	Low concern	Low concern	Low concern	110.2	110.9	95.9	3410.7	3095.5	6582.7	5937.9	1.9	2.0
Kentucky	2019	Low concern	Low concern	Low concern	Low concern	101.6	107.5	100.8	3146.5	3095.5	6383.3	5937.9	2.0	2.0
Louisiana	2019	High concern	High concern	Low concern	High concern	222.4	116.4	49.9	6885.0	3095.5	6914.4	5937.9	1.0	2.0

© 2024 Rodriguez MI et al. *JAMA Health Forum.*

Maine	2019	Low concern	Low concern	Low concern	Low concern	122.0	134.5	105.0	3776.7	3095.5	7984.0	5937.9	2.1	2.0
Maryland	2019	High concern	High concern	Low concern	Low concern	213.3	131.9	58.9	6603.3	3095.5	7832.5	5937.9	1.2	2.0
Massachusetts	2019	Medium concern	Medium concern	Low concern	Low concern	190.9	129.7	64.7	5910.1	3095.5	7699.6	5937.9	1.3	2.0
Michigan	2019	Low concern	Low concern	Low concern	Low concern	89.1	106.4	113.8	2757.2	3095.5	6315.2	5937.9	2.3	2.0
Minnesota	2019	Low concern	Low concern	Low concern	Low concern	111.6	126.4	108.0	3453.9	3095.5	7505.3	5937.9	2.2	2.0
Mississippi	2019	Low concern	Low concern	Low concern	Low concern	76.7	94.0	116.8	2375.0	3095.5	5582.5	5937.9	2.4	2.0
Missouri	2019	Low concern	Low concern	Low concern	Low concern	108.1	106.3	93.7	3346.7	3095.5	6312.8	5937.9	1.9	2.0
Montana	2019	Low concern	Low concern	Low concern	Low concern	84.5	86.6	97.6	2616.9	3095.5	5143.1	5937.9	2.0	2.0
Nebraska	2019	Low concern	Low concern	Low concern	Low concern	77.3	81.3	100.3	2392.7	3095.5	4830.4	5937.9	2.0	2.0
Nevada	2019	Medium concern	Medium concern	Medium concern	Low concern	65.5	72.8	105.9	2027.7	3095.5	4323.7	5937.9	2.1	2.0
New Hampshire	2019	Low concern	Low concern	Low concern	Low concern	85.4	94.0	104.9	2643.6	3095.5	5584.0	5937.9	2.1	2.0
New Jersey	2019	High concern	High concern	Low concern	High concern	248.5	129.6	49.7	7693.0	3095.5	7694.8	5937.9	1.0	2.0
New Mexico	2019	Low concern	Low concern	Low concern	Low concern	81.8	102.0	118.9	2532.2	3095.5	6057.9	5937.9	2.4	2.0
New York	2019	Low concern	Low concern	Low concern	Low concern	106.2	122.6	110.0	3288.8	3095.5	7279.4	5937.9	2.2	2.0
North Carolina	2019	Low concern	Low concern	Low concern	Low concern	76.9	91.7	113.7	2379.1	3095.5	5446.4	5937.9	2.3	2.0
North Dakota	2019	Low concern	Low concern	Low concern	Low concern	100.0	101.0	96.2	3095.5	3095.5	5996.3	5937.9	1.9	2.0
Ohio	2019	Medium concern	Low concern	Medium concern	Low concern	127.2	158.7	119.0	3936.2	3095.5	9425.2	5937.9	2.4	2.0
Oklahoma	2019	Medium concern	Medium concern	Medium concern	Low concern	66.4	72.4	103.9	2054.7	3095.5	4296.2	5937.9	2.1	2.0

© 2024 Rodriguez MI et al. *JAMA Health Forum.*

Oregon	2019	Low concern	Low concern	Low concern	Low concern	104.5	100.9	92.0	3235.8	3095.5	5994.1	5937.9	1.9	2.0
Pennsylvania	2019	Low concern	Low concern	Low concern	Low concern	117.1	112.4	91.5	3624.9	3095.5	6675.1	5937.9	1.8	2.0
Rhode Island	2019	Medium concern	Medium concern	Low concern	Low concern	191.4	138.2	68.8	5923.2	3095.5	8205.9	5937.9	1.4	2.0
South Carolina	2019	Low concern	Low concern	Low concern	Low concern	93.0	95.7	98.1	2877.9	3095.5	5680.2	5937.9	2.0	2.0
South Dakota	2019	Low concern	Low concern	Low concern	Low concern	113.4	79.4	66.8	3508.9	3095.5	4717.0	5937.9	1.3	2.0
Tennessee	2019	Low concern	Low concern	Low concern	Low concern	79.2	81.8	98.4	2450.9	3095.5	4855.5	5937.9	2.0	2.0
Texas	2019	Low concern	Low concern	Low concern	Low concern	102.4	122.9	114.4	3168.6	3095.5	7299.0	5937.9	2.3	2.0
Utah	2019	Medium concern	Medium concern	Low concern	Low concern	68.9	76.0	105.1	2132.5	3095.5	4513.1	5937.9	2.1	2.0
Vermont	2019	Low concern	Low concern	Low concern	Low concern	146.9	105.5	68.4	4547.0	3095.5	6261.8	5937.9	1.4	2.0
Virginia	2019	Low concern	Low concern	Low concern	Low concern	106.2	80.3	72.1	3287.1	3095.5	4767.7	5937.9	1.5	2.0
Washington	2019	Low concern	Low concern	Low concern	Low concern	91.6	98.6	102.5	2836.3	3095.5	5852.7	5937.9	2.1	2.0
West Virginia	2019	Medium concern	Medium concern	Medium concern	Low concern	61.6	71.2	110.1	1907.9	3095.5	4228.3	5937.9	2.2	2.0
Wisconsin	2019	Low concern	Low concern	Low concern	Low concern	101.5	100.0	93.9	3142.9	3095.5	5937.9	5937.9	1.9	2.0
Wyoming	2019	Low concern	Low concern	Low concern	Low concern	78.1	88.6	108.1	2417.7	3095.5	5258.8	5937.9	2.2	2.0

© 2024 Rodriguez MI et al. *JAMA Health Forum.*

Supplement eTable 3. RX claims volume data quality assessment for eligible women ages 18 to 44 who gave birth in 2016-2019

State	Data Year	DQ Assessment	Assessment of RX File Header Record Volume	Assessment of RX File Line Record Volume	Assessment of Avg RX Line Records per Header	RX File Header Records per 1,000 Enrolled Months as % of National Median	RX File Line Records per 1,000 Enrolled Months as % of National Median	Avg RX File Line Records per Header Record as % of National Median	RX File Header Records per 1,000 Enrolled Months	National Median: Header Records per 1,000 Enrolled Months in RX File	RX File Line Records per 1,000 Enrolled Months	National Median: Line Records per 1,000 Enrolled Months in RX File	Avg RX File Line Records per Header Record	National Median: Avg Line Records per Header in RX File
Alabama	2016	Medium concern	Medium concern	Medium concern	Low concern	68.4	68.0	100.0	583.6	853.3	584.1	858.4	1.0	1.0
Alaska	2016	Medium concern	Medium concern	Medium concern	Low concern	68.4	68.1	100.1	583.5	853.3	584.8	858.4	1.0	1.0
Arizona	2016	Low concern	Low concern	Low concern	Low concern	90.2	89.7	99.9	770.1	853.3	770.1	858.4	1.0	1.0
Arkansas	2016	High concern	High concern	High concern	Low concern	25.0	24.9	100.0	213.7	853.3	214.0	858.4	1.0	1.0
California	2016	Low concern	Low concern	Low concern	Low concern	94.7	94.2	99.9	808.4	853.3	808.4	858.4	1.0	1.0
Colorado	2016	Medium concern	Medium concern	Medium concern	Low concern	67.7	67.4	100.0	577.8	853.3	578.6	858.4	1.0	1.0
Connecticut	2016	Low concern	Low concern	Low concern	Low concern	115.1	114.7	100.1	982.6	853.3	984.8	858.4	1.0	1.0
Delaware	2016	Low concern	Low concern	Low concern	Low concern	103.3	102.9	100.1	881.9	853.3	883.3	858.4	1.0	1.0
District of Columbia	2016	Low concern	Low concern	Low concern	Low concern	100.9	100.4	100.0	861.1	853.3	861.7	858.4	1.0	1.0
Florida	2016	Medium concern	Medium concern	Medium concern	Low concern	73.4	73.0	99.9	626.4	853.3	626.4	858.4	1.0	1.0

© 2024 Rodriguez MI et al. *JAMA Health Forum.*

Georgia	2016	Low concern	Low concern	Low concern	Low concern	112.1	111.4	99.9	956.5	853.3	956.5	858.4	1.0	1.0
Hawaii	2016	Low concern	Low concern	Low concern	Low concern	76.7	76.3	99.9	654.8	853.3	654.8	858.4	1.0	1.0
Idaho	2016	Low concern	Low concern	Low concern	Low concern	99.0	98.5	99.9	845.2	853.3	845.2	858.4	1.0	1.0
Illinois	2016	Low concern	Low concern	Low concern	Low concern	93.5	93.7	100.6	798.1	853.3	804.0	858.4	1.0	1.0
Indiana	2016	Low concern	Low concern	Low concern	Low concern	98.2	98.3	100.6	838.0	853.3	843.8	858.4	1.0	1.0
Iowa	2016	Low concern	Low concern	Low concern	Low concern	96.9	96.3	99.9	826.6	853.3	826.5	858.4	1.0	1.0
Kansas	2016	Low concern	Low concern	Low concern	Low concern	96.1	97.4	101.8	820.3	853.3	835.8	858.4	1.0	1.0
Kentucky	2016	Low concern	Low concern	Low concern	Low concern	132.2	131.9	100.3	1128.2	853.3	1132.7	858.4	1.0	1.0
Louisiana	2016	Low concern	Low concern	Low concern	Low concern	100.6	100.0	99.9	858.4	853.3	858.4	858.4	1.0	1.0
Maine	2016	Low concern	Low concern	Low concern	Low concern	117.1	116.6	100.1	999.0	853.3	1001.2	858.4	1.0	1.0
Maryland	2016	Low concern	Low concern	Low concern	Low concern	118.4	117.7	99.9	1010.3	853.3	1010.3	858.4	1.0	1.0
Massachusetts	2016	Low concern	Low concern	Low concern	Low concern	114.4	107.8	94.7	975.9	853.3	925.2	858.4	0.9	1.0
Michigan	2016	Low concern	Low concern	Low concern	Low concern	118.8	118.1	99.9	1014.1	853.3	1014.0	858.4	1.0	1.0
Minnesota	2016	Low concern	Low concern	Low concern	Low concern	134.0	121.1	90.8	1143.1	853.3	1039.5	858.4	0.9	1.0
Mississippi	2016	Unusable	Low concern	Unusable	Unusable	93.8	8.0	8.5	800.4	853.3	68.4	858.4	0.1	1.0
Missouri	2016	Low concern	Low concern	Low concern	Low concern	129.1	128.3	99.9	1101.4	853.3	1101.4	858.4	1.0	1.0
Montana	2016	Low concern	Low concern	Low concern	Low concern	77.3	76.8	99.9	659.2	853.3	659.2	858.4	1.0	1.0
Nebraska	2016	Low concern	Low concern	Low concern	Low concern	112.9	112.2	99.9	963.1	853.3	963.1	858.4	1.0	1.0

© 2024 Rodriguez MI et al. *JAMA Health Forum.*

Nevada	2016	Low concern	Low concern	Low concern	Low concern	87.0	86.5	99.9	742.1	853.3	742.2	858.4	1.0	1.0
New Hampshire	2016	Low concern	Low concern	Low concern	Low concern	90.2	90.1	100.3	770.0	853.3	773.2	858.4	1.0	1.0
New Jersey	2016	Low concern	Low concern	Low concern	Low concern	119.9	119.6	100.3	1022.9	853.3	1026.6	858.4	1.0	1.0
New Mexico	2016	Low concern	Low concern	Low concern	Low concern	84.2	84.3	100.6	718.7	853.3	723.7	858.4	1.0	1.0
New York	2016	Low concern	Low concern	Low concern	Low concern	122.2	121.5	99.9	1042.7	853.3	1042.7	858.4	1.0	1.0
North Carolina	2016	High concern	High concern	High concern	Low concern	34.3	34.2	100.1	293.1	853.3	293.7	858.4	1.0	1.0
North Dakota	2016	Low concern	Low concern	Low concern	Low concern	77.5	78.1	101.2	661.6	853.3	670.4	858.4	1.0	1.0
Ohio	2016	Low concern	Low concern	Low concern	Low concern	142.7	142.4	100.3	1217.4	853.3	1222.3	858.4	1.0	1.0
Oklahoma	2016	Low concern	Low concern	Low concern	Low concern	100.0	100.8	101.3	853.3	853.3	865.6	858.4	1.0	1.0
Oregon	2016	Low concern	Low concern	Low concern	Low concern	90.6	90.3	100.2	773.3	853.3	775.4	858.4	1.0	1.0
Pennsylvania	2016	Low concern	Low concern	Low concern	Low concern	142.7	142.4	100.3	1218.0	853.3	1222.8	858.4	1.0	1.0
Rhode Island	2016	Low concern	Low concern	Low concern	Low concern	133.3	132.5	99.9	1137.4	853.3	1137.4	858.4	1.0	1.0
South Carolina	2016	Low concern	Low concern	Low concern	Low concern	80.3	79.8	99.9	684.9	853.3	684.9	858.4	1.0	1.0
South Dakota	2016	Low concern	Low concern	Low concern	Low concern	79.8	79.6	100.2	681.0	853.3	683.0	858.4	1.0	1.0
Tennessee	2016	Low concern	Low concern	Low concern	Low concern	109.1	109.8	101.2	931.0	853.3	942.9	858.4	1.0	1.0
Texas	2016	Low concern	Low concern	Low concern	Low concern	105.7	105.1	99.9	901.6	853.3	901.9	858.4	1.0	1.0
Utah	2016	Low concern	Low concern	Low concern	Low concern	116.7	116.8	100.6	995.6	853.3	1002.9	858.4	1.0	1.0
Vermont	2016	Low concern	Low concern	Low concern	Low concern	134.1	133.3	99.9	1144.2	853.3	1144.2	858.4	1.0	1.0

© 2024 Rodriguez MI et al. *JAMA Health Forum.*

Virginia	2016	Low concern	Low concern	Low concern	Low concern	94.4	94.4	100.5	805.5	853.3	810.6	858.4	1.0	1.0
Washington	2016	Low concern	Low concern	Low concern	Low concern	104.5	104.1	100.1	891.9	853.3	893.4	858.4	1.0	1.0
West Virginia	2016	Low concern	Low concern	Low concern	Low concern	115.9	115.2	99.9	989.0	853.3	988.8	858.4	1.0	1.0
Wisconsin	2016	Low concern	Low concern	Low concern	Low concern	103.0	103.0	100.5	879.1	853.3	884.1	858.4	1.0	1.0
Wyoming	2016	Medium concern	Medium concern	Medium concern	Low concern	73.3	73.0	100.1	625.3	853.3	626.5	858.4	1.0	1.0
Alabama	2017	Medium concern	Medium concern	Medium concern	Low concern	66.0	65.4	100.0	572.6	868.0	573.3	876.2	1.0	1.0
Alaska	2017	Medium concern	Medium concern	Medium concern	Low concern	63.8	63.3	100.1	553.6	868.0	554.9	876.2	1.0	1.0
Arizona	2017	Low concern	Low concern	Low concern	Low concern	92.2	91.3	99.9	800.2	868.0	800.2	876.2	1.0	1.0
Arkansas	2017	High concern	High concern	High concern	Low concern	44.0	43.7	100.0	382.1	868.0	382.5	876.2	1.0	1.0
California	2017	Low concern	Low concern	Low concern	Low concern	96.2	95.3	99.9	834.8	868.0	834.8	876.2	1.0	1.0
Colorado	2017	Medium concern	Medium concern	Medium concern	Low concern	64.3	63.8	100.0	557.8	868.0	558.7	876.2	1.0	1.0
Connecticut	2017	Low concern	Low concern	Low concern	Low concern	115.5	114.8	100.2	1002.6	868.0	1006.1	876.2	1.0	1.0
Delaware	2017	Low concern	Low concern	Low concern	Low concern	84.7	84.0	100.0	735.0	868.0	736.2	876.2	1.0	1.0
District of Columbia	2017	Low concern	Low concern	Low concern	Low concern	89.3	88.5	99.9	774.8	868.0	775.0	876.2	1.0	1.0
Florida	2017	High concern	High concern	High concern	Low concern	38.8	38.4	99.9	336.8	868.0	336.8	876.2	1.0	1.0
Georgia	2017	Low concern	Low concern	Low concern	Low concern	103.2	102.2	99.9	895.5	868.0	895.5	876.2	1.0	1.0
Hawaii	2017	Low concern	Low concern	Low concern	Low concern	79.2	78.4	99.9	687.0	868.0	687.0	876.2	1.0	1.0
Idaho	2017	Low concern	Low concern	Low concern	Low concern	102.2	101.3	99.9	887.2	868.0	887.2	876.2	1.0	1.0

© 2024 Rodriguez MI et al. *JAMA Health Forum.*

Illinois	2017	Low concern	Low concern	Low concern	Low concern	92.4	92.1	100.5	802.0	868.0	807.2	876.2	1.0	1.0
Indiana	2017	Low concern	Low concern	Low concern	Low concern	96.1	95.5	100.1	834.4	868.0	836.5	876.2	1.0	1.0
Iowa	2017	Low concern	Low concern	Low concern	Low concern	90.0	89.2	99.9	781.6	868.0	781.6	876.2	1.0	1.0
Kansas	2017	Low concern	Low concern	Low concern	Low concern	102.0	102.5	101.3	885.2	868.0	898.0	876.2	1.0	1.0
Kentucky	2017	Low concern	Low concern	Low concern	Low concern	134.6	133.8	100.2	1168.5	868.0	1172.6	876.2	1.0	1.0
Louisiana	2017	Low concern	Low concern	Low concern	Low concern	105.5	104.5	99.9	915.7	868.0	915.7	876.2	1.0	1.0
Maine	2017	Low concern	Low concern	Low concern	Low concern	110.0	109.1	100.1	954.5	868.0	956.3	876.2	1.0	1.0
Maryland	2017	Low concern	Low concern	Low concern	Low concern	115.3	114.2	99.9	1000.5	868.0	1000.5	876.2	1.0	1.0
Massachusetts	2017	Low concern	Low concern	Low concern	Low concern	118.7	117.8	100.0	1030.7	868.0	1031.8	876.2	1.0	1.0
Michigan	2017	Low concern	Low concern	Low concern	Low concern	124.9	123.7	99.9	1084.3	868.0	1084.2	876.2	1.0	1.0
Minnesota	2017	Low concern	Low concern	Low concern	Low concern	122.0	114.7	94.8	1058.8	868.0	1005.0	876.2	0.9	1.0
Mississippi	2017	Low concern	Low concern	Low concern	Low concern	91.9	91.0	99.9	797.5	868.0	797.5	876.2	1.0	1.0
Missouri	2017	Low concern	Low concern	Low concern	Low concern	129.9	128.7	99.9	1127.4	868.0	1127.4	876.2	1.0	1.0
Montana	2017	Low concern	Low concern	Low concern	Low concern	92.9	92.1	99.9	806.6	868.0	806.6	876.2	1.0	1.0
Nebraska	2017	Low concern	Low concern	Low concern	Low concern	119.0	120.8	102.3	1033.0	868.0	1058.3	876.2	1.0	1.0
Nevada	2017	Low concern	Low concern	Low concern	Low concern	90.8	89.9	99.9	787.8	868.0	787.8	876.2	1.0	1.0
New Hampshire	2017	Low concern	Low concern	Low concern	Low concern	88.6	87.9	100.1	768.7	868.0	770.2	876.2	1.0	1.0
New Jersey	2017	Low concern	Low concern	Low concern	Low concern	121.3	120.7	100.3	1052.8	868.0	1057.7	876.2	1.0	1.0

© 2024 Rodriguez MI et al. *JAMA Health Forum.*

New Mexico	2017	Low concern	Low concern	Low concern	Low concern	80.6	80.8	101.1	699.4	868.0	707.7	876.2	1.0	1.0
New York	2017	Low concern	Low concern	Low concern	Low concern	124.1	123.0	99.9	1077.4	868.0	1077.7	876.2	1.0	1.0
North Carolina	2017	High concern	High concern	High concern	Low concern	19.1	19.0	100.0	165.9	868.0	166.2	876.2	1.0	1.0
North Dakota	2017	Low concern	Low concern	Low concern	Low concern	82.6	83.6	102.1	716.7	868.0	732.6	876.2	1.0	1.0
Ohio	2017	Low concern	Low concern	Low concern	Low concern	141.6	141.0	100.3	1229.5	868.0	1235.0	876.2	1.0	1.0
Oklahoma	2017	Low concern	Low concern	Low concern	Low concern	102.2	102.9	101.5	886.9	868.0	901.3	876.2	1.0	1.0
Oregon	2017	Low concern	Low concern	Low concern	Low concern	89.5	88.9	100.2	777.2	868.0	779.3	876.2	1.0	1.0
Pennsylvania	2017	Low concern	Low concern	Low concern	Low concern	136.9	136.2	100.3	1188.4	868.0	1193.0	876.2	1.0	1.0
Rhode Island	2017	Low concern	Low concern	Low concern	Low concern	133.7	132.4	99.9	1160.2	868.0	1160.2	876.2	1.0	1.0
South Carolina	2017	Low concern	Low concern	Low concern	Low concern	82.2	81.4	99.9	713.5	868.0	713.5	876.2	1.0	1.0
South Dakota	2017	Medium concern	Low concern	Medium concern	Low concern	75.0	74.5	100.1	651.4	868.0	653.0	876.2	1.0	1.0
Tennessee	2017	Low concern	Low concern	Low concern	Low concern	101.7	101.8	100.9	883.1	868.0	892.0	876.2	1.0	1.0
Texas	2017	Low concern	Low concern	Low concern	Low concern	103.0	102.1	99.9	894.3	868.0	894.6	876.2	1.0	1.0
Utah	2017	Low concern	Low concern	Low concern	Low concern	118.4	118.2	100.6	1028.0	868.0	1035.5	876.2	1.0	1.0
Vermont	2017	Low concern	Low concern	Low concern	Low concern	132.6	131.4	99.9	1151.1	868.0	1151.1	876.2	1.0	1.0
Virginia	2017	Low concern	Low concern	Low concern	Low concern	91.3	91.4	100.9	792.3	868.0	800.4	876.2	1.0	1.0
Washington	2017	Low concern	Low concern	Low concern	Low concern	103.2	102.4	100.0	896.2	868.0	897.0	876.2	1.0	1.0
West Virginia	2017	Low concern	Low concern	Low concern	Low concern	122.0	120.9	99.9	1059.3	868.0	1059.3	876.2	1.0	1.0

© 2024 Rodriguez MI et al. *JAMA Health Forum.*

Wisconsin	2017	Low concern	Low concern	Low concern	Low concern	100.0	100.0	100.8	868.0	868.0	876.2	876.2	1.0	1.0
Wyoming	2017	Low concern	Low concern	Low concern	Low concern	89.2	88.6	100.1	774.5	868.0	776.3	876.2	1.0	1.0
Alabama	2018	Medium concern	Medium concern	Medium concern	Low concern	61.3	61.2	100.0	509.4	831.4	509.9	832.6	1.0	1.0
Alaska	2018	Medium concern	Medium concern	Medium concern	Low concern	67.5	67.6	100.1	561.0	831.4	562.5	832.6	1.0	1.0
Arizona	2018	Low concern	Low concern	Low concern	Low concern	96.2	96.0	99.9	799.5	831.4	799.5	832.6	1.0	1.0
Arkansas	2018	Medium concern	Medium concern	Medium concern	Low concern	60.9	60.8	99.9	506.2	831.4	506.4	832.6	1.0	1.0
California	2018	Low concern	Low concern	Low concern	Low concern	102.6	102.4	99.9	853.0	831.4	853.0	832.6	1.0	1.0
Colorado	2018	Medium concern	Medium concern	Medium concern	Low concern	64.5	64.6	100.1	536.5	831.4	537.6	832.6	1.0	1.0
Connecticut	2018	Low concern	Low concern	Low concern	Low concern	117.0	117.3	100.3	972.6	831.4	976.5	832.6	1.0	1.0
Delaware	2018	Low concern	Low concern	Low concern	Low concern	100.0	100.0	100.0	831.4	831.4	832.6	832.6	1.0	1.0
District of Columbia	2018	Low concern	Low concern	Low concern	Low concern	94.4	94.3	99.9	784.8	831.4	784.9	832.6	1.0	1.0
Florida	2018	Low concern	Low concern	Low concern	Low concern	83.1	83.0	99.9	690.9	831.4	690.9	832.6	1.0	1.0
Georgia	2018	Low concern	Low concern	Low concern	Low concern	97.7	97.6	99.9	812.5	831.4	812.5	832.6	1.0	1.0
Hawaii	2018	Low concern	Low concern	Low concern	Low concern	79.9	79.8	99.9	664.3	831.4	664.3	832.6	1.0	1.0
Idaho	2018	Low concern	Low concern	Low concern	Low concern	106.7	106.5	99.9	886.7	831.4	886.7	832.6	1.0	1.0
Illinois	2018	Low concern	Low concern	Low concern	Low concern	89.8	90.3	100.7	746.3	831.4	752.2	832.6	1.0	1.0
Indiana	2018	Low concern	Low concern	Low concern	Low concern	107.7	107.8	100.1	895.3	831.4	897.3	832.6	1.0	1.0
Iowa	2018	Low concern	Low concern	Low concern	Low concern	95.4	95.2	99.9	792.8	831.4	792.8	832.6	1.0	1.0

© 2024 Rodriguez MI et al. *JAMA Health Forum.*

Kansas	2018	Low concern	Low concern	Low concern	Low concern	97.6	98.7	101.1	811.7	831.4	821.4	832.6	1.0	1.0
Kentucky	2018	Low concern	Low concern	Low concern	Low concern	142.5	142.7	100.2	1184.9	831.4	1188.3	832.6	1.0	1.0
Louisiana	2018	Low concern	Low concern	Low concern	Low concern	115.4	115.3	99.9	959.7	831.4	959.7	832.6	1.0	1.0
Maine	2018	Low concern	Low concern	Low concern	Low concern	105.6	105.6	100.1	877.7	831.4	879.4	832.6	1.0	1.0
Maryland	2018	Low concern	Low concern	Low concern	Low concern	123.8	123.6	99.9	1029.3	831.4	1029.3	832.6	1.0	1.0
Massachusetts	2018	Low concern	Low concern	Low concern	Low concern	131.3	131.3	100.0	1091.9	831.4	1093.1	832.6	1.0	1.0
Michigan	2018	Low concern	Low concern	Low concern	Low concern	127.7	127.5	99.9	1061.3	831.4	1061.3	832.6	1.0	1.0
Minnesota	2018	Low concern	Low concern	Low concern	Low concern	127.6	122.7	96.2	1060.8	831.4	1021.2	832.6	1.0	1.0
Mississippi	2018	Low concern	Low concern	Low concern	Low concern	94.5	94.4	99.9	786.1	831.4	786.1	832.6	1.0	1.0
Missouri	2018	Low concern	Low concern	Low concern	Low concern	132.8	132.6	99.9	1104.3	831.4	1104.3	832.6	1.0	1.0
Montana	2018	Low concern	Low concern	Low concern	Low concern	93.4	93.3	99.9	776.8	831.4	776.8	832.6	1.0	1.0
Nebraska	2018	Low concern	Low concern	Low concern	Low concern	123.1	123.1	100.0	1023.4	831.4	1024.9	832.6	1.0	1.0
Nevada	2018	Low concern	Low concern	Low concern	Low concern	83.3	83.2	99.9	692.7	831.4	692.8	832.6	1.0	1.0
New Hampshire	2018	Low concern	Low concern	Low concern	Low concern	98.2	98.3	100.1	816.7	831.4	818.5	832.6	1.0	1.0
New Jersey	2018	Low concern	Low concern	Low concern	Low concern	128.3	128.7	100.4	1066.7	831.4	1071.8	832.6	1.0	1.0
New Mexico	2018	Low concern	Low concern	Low concern	Low concern	85.7	86.5	101.0	712.2	831.4	720.1	832.6	1.0	1.0
New York	2018	Low concern	Low concern	Low concern	Low concern	135.8	135.7	99.9	1129.3	831.4	1129.6	832.6	1.0	1.0
North Carolina	2018	High concern	High concern	High concern	Low concern	18.4	18.4	100.0	152.6	831.4	152.8	832.6	1.0	1.0

© 2024 Rodriguez MI et al. *JAMA Health Forum.*

North Dakota	2018	Low concern	Low concern	Low concern	Low concern	88.4	89.1	100.8	734.7	831.4	741.5	832.6	1.0	1.0
Ohio	2018	Medium concern	Medium concern	Medium concern	Low concern	150.8	151.5	100.5	1254.0	831.4	1261.7	832.6	1.0	1.0
Oklahoma	2018	Low concern	Low concern	Low concern	Low concern	101.9	103.5	101.6	847.4	831.4	861.9	832.6	1.0	1.0
Oregon	2018	Low concern	Low concern	Low concern	Low concern	96.8	96.9	100.1	805.0	831.4	806.8	832.6	1.0	1.0
Pennsylvania	2018	Low concern	Low concern	Low concern	Low concern	137.5	137.8	100.2	1143.3	831.4	1147.0	832.6	1.0	1.0
Rhode Island	2018	High concern	High concern	High concern	Low concern	256.9	256.5	99.9	2135.7	831.4	2135.7	832.6	1.0	1.0
South Carolina	2018	Low concern	Low concern	Low concern	Low concern	81.8	81.7	99.9	680.3	831.4	680.3	832.6	1.0	1.0
South Dakota	2018	Low concern	Low concern	Low concern	Low concern	77.6	77.7	100.2	644.8	831.4	646.7	832.6	1.0	1.0
Tennessee	2018	Low concern	Low concern	Low concern	Low concern	98.0	98.2	100.3	814.5	831.4	817.9	832.6	1.0	1.0
Texas	2018	Low concern	Low concern	Low concern	Low concern	103.8	103.7	99.9	863.3	831.4	863.5	832.6	1.0	1.0
Utah	2018	Low concern	Low concern	Low concern	Low concern	116.3	116.9	100.5	967.2	831.4	973.6	832.6	1.0	1.0
Vermont	2018	Low concern	Low concern	Low concern	Low concern	127.9	127.7	99.9	1063.2	831.4	1063.2	832.6	1.0	1.0
Virginia	2018	Low concern	Low concern	Low concern	Low concern	96.5	97.0	100.6	802.0	831.4	807.7	832.6	1.0	1.0
Washington	2018	Low concern	Low concern	Low concern	Low concern	108.0	108.0	100.0	898.2	831.4	899.0	832.6	1.0	1.0
West Virginia	2018	Low concern	Low concern	Low concern	Low concern	133.9	133.7	99.9	1113.2	831.4	1113.2	832.6	1.0	1.0
Wisconsin	2018	Low concern	Low concern	Low concern	Low concern	105.1	105.4	100.4	873.5	831.4	877.7	832.6	1.0	1.0
Wyoming	2018	Low concern	Low concern	Low concern	Low concern	93.5	93.5	100.1	777.0	831.4	778.6	832.6	1.0	1.0
Alabama	2019	High concern	High concern	High concern	Low concern	43.8	43.8	100.0	380.1	868.6	380.5	868.6	1.0	1.0

© 2024 Rodriguez MI et al. *JAMA Health Forum.*

Alaska	2019	Medium concern	Medium concern	Medium concern	Low concern	67.4	67.5	100.1	585.0	868.6	586.3	868.6	1.0	1.0
Arizona	2019	Low concern	Low concern	Low concern	Low concern	96.0	96.0	99.9	834.1	868.6	834.1	868.6	1.0	1.0
Arkansas	2019	Medium concern	Medium concern	Medium concern	Low concern	59.2	59.2	100.0	514.1	868.6	514.4	868.6	1.0	1.0
California	2019	Low concern	Low concern	Low concern	Low concern	100.0	100.0	99.9	868.6	868.6	868.6	868.6	1.0	1.0
Colorado	2019	Medium concern	Medium concern	Medium concern	Low concern	62.8	62.9	100.1	545.4	868.6	546.6	868.6	1.0	1.0
Connecticut	2019	Low concern	Low concern	Low concern	Low concern	114.2	114.5	100.2	991.6	868.6	994.5	868.6	1.0	1.0
Delaware	2019	Low concern	Low concern	Low concern	Low concern	121.3	121.4	100.0	1053.7	868.6	1054.8	868.6	1.0	1.0
District of Columbia	2019	Low concern	Low concern	Low concern	Low concern	98.4	98.4	99.9	854.8	868.6	855.1	868.6	1.0	1.0
Florida	2019	Low concern	Low concern	Low concern	Low concern	78.1	78.1	99.9	678.6	868.6	678.6	868.6	1.0	1.0
Georgia	2019	Low concern	Low concern	Low concern	Low concern	93.9	93.9	99.9	815.8	868.6	815.8	868.6	1.0	1.0
Hawaii	2019	Medium concern	Medium concern	Medium concern	Low concern	71.2	71.2	99.9	618.8	868.6	618.8	868.6	1.0	1.0
Idaho	2019	Low concern	Low concern	Low concern	Low concern	102.2	102.2	99.9	887.6	868.6	887.6	868.6	1.0	1.0
Illinois	2019	Low concern	Low concern	Low concern	Low concern	89.7	90.9	101.3	778.8	868.6	789.6	868.6	1.0	1.0
Indiana	2019	Low concern	Low concern	Low concern	Low concern	104.2	104.3	100.0	904.8	868.6	905.8	868.6	1.0	1.0
Iowa	2019	Low concern	Low concern	Low concern	Low concern	88.8	88.8	99.9	771.5	868.6	771.5	868.6	1.0	1.0
Kansas	2019	Low concern	Low concern	Low concern	Low concern	93.1	94.6	101.5	809.0	868.6	822.0	868.6	1.0	1.0
Kentucky	2019	Low concern	Low concern	Low concern	Low concern	137.1	137.5	100.2	1190.9	868.6	1194.1	868.6	1.0	1.0
Louisiana	2019	Low concern	Low concern	Low concern	Low concern	108.5	108.5	99.9	942.4	868.6	942.4	868.6	1.0	1.0

© 2024 Rodriguez MI et al. *JAMA Health Forum.*

Maine	2019	Low concern	Low concern	Low concern	Low concern	100.2	100.4	100.1	870.5	868.6	872.3	868.6	1.0	1.0
Maryland	2019	Low concern	Low concern	Low concern	Low concern	120.8	120.8	99.9	1049.5	868.6	1049.5	868.6	1.0	1.0
Massachusetts	2019	Low concern	Low concern	Low concern	Low concern	130.0	130.2	100.0	1129.5	868.6	1131.0	868.6	1.0	1.0
Michigan	2019	Low concern	Low concern	Low concern	Low concern	116.7	116.7	99.9	1013.4	868.6	1013.4	868.6	1.0	1.0
Minnesota	2019	Low concern	Low concern	Low concern	Low concern	121.4	121.6	100.0	1054.4	868.6	1056.1	868.6	1.0	1.0
Mississippi	2019	Low concern	Low concern	Low concern	Low concern	88.8	88.8	99.9	771.1	868.6	771.2	868.6	1.0	1.0
Missouri	2019	Low concern	Low concern	Low concern	Low concern	136.0	136.0	99.9	1181.6	868.6	1181.6	868.6	1.0	1.0
Montana	2019	Low concern	Low concern	Low concern	Low concern	99.1	99.1	99.9	860.9	868.6	860.9	868.6	1.0	1.0
Nebraska	2019	Low concern	Low concern	Low concern	Low concern	106.2	106.3	100.0	922.2	868.6	923.2	868.6	1.0	1.0
Nevada	2019	Low concern	Low concern	Low concern	Low concern	96.2	96.3	100.0	836.0	868.6	836.7	868.6	1.0	1.0
New Hampshire	2019	Low concern	Low concern	Low concern	Low concern	106.2	106.4	100.1	922.4	868.6	924.4	868.6	1.0	1.0
New Jersey	2019	Low concern	Low concern	Low concern	Low concern	124.2	124.8	100.4	1078.6	868.6	1083.6	868.6	1.0	1.0
New Mexico	2019	Low concern	Low concern	Low concern	Low concern	81.8	82.7	101.0	710.7	868.6	718.4	868.6	1.0	1.0
New York	2019	Low concern	Low concern	Low concern	Low concern	133.6	133.6	99.9	1160.0	868.6	1160.3	868.6	1.0	1.0
North Carolina	2019	High concern	High concern	High concern	Low concern	16.4	16.5	100.1	142.6	868.6	143.0	868.6	1.0	1.0
North Dakota	2019	Low concern	Low concern	Low concern	Low concern	78.6	79.0	100.4	682.3	868.6	685.8	868.6	1.0	1.0
Ohio	2019	Low concern	Low concern	Low concern	Low concern	145.9	146.7	100.5	1266.9	868.6	1274.5	868.6	1.0	1.0
Oklahoma	2019	Low concern	Low concern	Low concern	Low concern	94.7	96.3	101.6	822.6	868.6	836.7	868.6	1.0	1.0

© 2024 Rodriguez MI et al. *JAMA Health Forum.*

Oregon	2019	Low concern	Low concern	Low concern	Low concern	96.0	96.1	100.0	834.1	868.6	834.9	868.6	1.0	1.0
Pennsylvania	2019	Low concern	Low concern	Low concern	Low concern	133.2	133.6	100.2	1157.1	868.6	1160.3	868.6	1.0	1.0
Rhode Island	2019	High concern	High concern	High concern	Low concern	214.6	214.6	99.9	1864.3	868.6	1864.3	868.6	1.0	1.0
South Carolina	2019	Low concern	Low concern	Low concern	Low concern	77.3	77.3	99.9	671.6	868.6	671.6	868.6	1.0	1.0
South Dakota	2019	Low concern	Low concern	Low concern	Low concern	81.8	81.9	100.1	710.2	868.6	711.7	868.6	1.0	1.0
Tennessee	2019	Low concern	Low concern	Low concern	Low concern	86.2	86.5	100.2	749.0	868.6	751.6	868.6	1.0	1.0
Texas	2019	Low concern	Low concern	Low concern	Low concern	94.8	94.8	99.9	823.3	868.6	823.5	868.6	1.0	1.0
Utah	2019	Low concern	Low concern	Low concern	Low concern	115.5	116.2	100.6	1002.9	868.6	1009.7	868.6	1.0	1.0
Vermont	2019	Low concern	Low concern	Low concern	Low concern	127.6	127.6	99.9	1108.1	868.6	1108.1	868.6	1.0	1.0
Virginia	2019	Low concern	Low concern	Low concern	Low concern	105.2	105.5	100.1	914.0	868.6	916.1	868.6	1.0	1.0
Washington	2019	Low concern	Low concern	Low concern	Low concern	110.0	110.1	100.0	955.5	868.6	956.3	868.6	1.0	1.0
West Virginia	2019	Low concern	Low concern	Low concern	Low concern	130.3	130.1	99.8	1131.6	868.6	1130.4	868.6	1.0	1.0
Wisconsin	2019	Low concern	Low concern	Low concern	Low concern	101.1	101.5	100.3	878.0	868.6	881.4	868.6	1.0	1.0
Wyoming	2019	Low concern	Low concern	Low concern	Low concern	81.1	81.3	100.2	704.2	868.6	706.1	868.6	1.0	1.0

© 2024 Rodriguez MI et al. *JAMA Health Forum.*

Supplement Section 3. Staggered Difference-in-differences model specification

We assessed the association of the policy with each of our outcomes using a staggered difference-in-differences model as described in Sun and Abraham, 2021[7]. We modeled our outcome using a linear probability model with the following form:

$$Y_{ist} = \beta_0 + \vec{\beta}_1 \overrightarrow{Wave}_{is} + \vec{\beta}_2 \overrightarrow{Month}_{it} + \vec{\beta}_3 \overrightarrow{Wave}_{is} \overrightarrow{MonthsFromPolicy}_{ist} + \vec{\beta}_4 \vec{X}_{ist} + \varepsilon_{ist}$$

Where Y_{ist} represents the outcome Y for delivery i in state s and month t, β_0 is the intercept, $\vec{\beta}_1 \overrightarrow{Wave}_{is}$ is a vector of policy implementation wave fixed effects to account for baseline differences between implementation wave, and $\vec{\beta}_2 \overrightarrow{Month}_{it}$ is a vector of month fixed effects to account for nationwide secular time trends. Our coefficients of interest are $\vec{\beta}_3$, representing the policy effect estimates associated with each policy in each month pre- and post-implementation. $\overrightarrow{Wave}_{is} \overrightarrow{MonthsFromPolicy}_{ics}$ is a vector of interaction terms between the policy implementation wave of the state the policy occurred in $\overrightarrow{Wave}_{is}$ and the months since the policy implementation in that wave. These interaction terms are only created for months that have study data, and we exclude terms for the period three months prior to the policy as a reference group to avoid overparameterizing the model and to allow for short term anticipatory effects prior to the policy implementation. For example, wave 1 implemented the policy 5 months after the start of the study period, and thus we will create interaction terms for months fou and two through one months prior to study and for months 0 through 33 months following the implementation of the policy. In total, the model contains 113 such interaction terms. $\vec{\beta}_4 \vec{X}_{ist}$ controls for the effect of patient and state characteristics $\vec{X}_{ist}$, and ε_{ist} represents the normally distributed error term.

Aggregate measures of treatment effect at the population level, the month-level, and the policy implementation wave level were estimated by averaging the interaction term coefficients weighted by the relative population size captured by each month and wave combination. We used a post-hoc Huber-White cluster robust standard error correction to cluster the standard errors at the state level.

Supplemental Section 4. Unadjusted time trends for secondary outcomes

Supplemental eFigure 2. Unadjusted monthly rates of Interval LARC over time stratified by policy implementation wave

© 2024 Rodriguez MI et al. *JAMA Health Forum.*

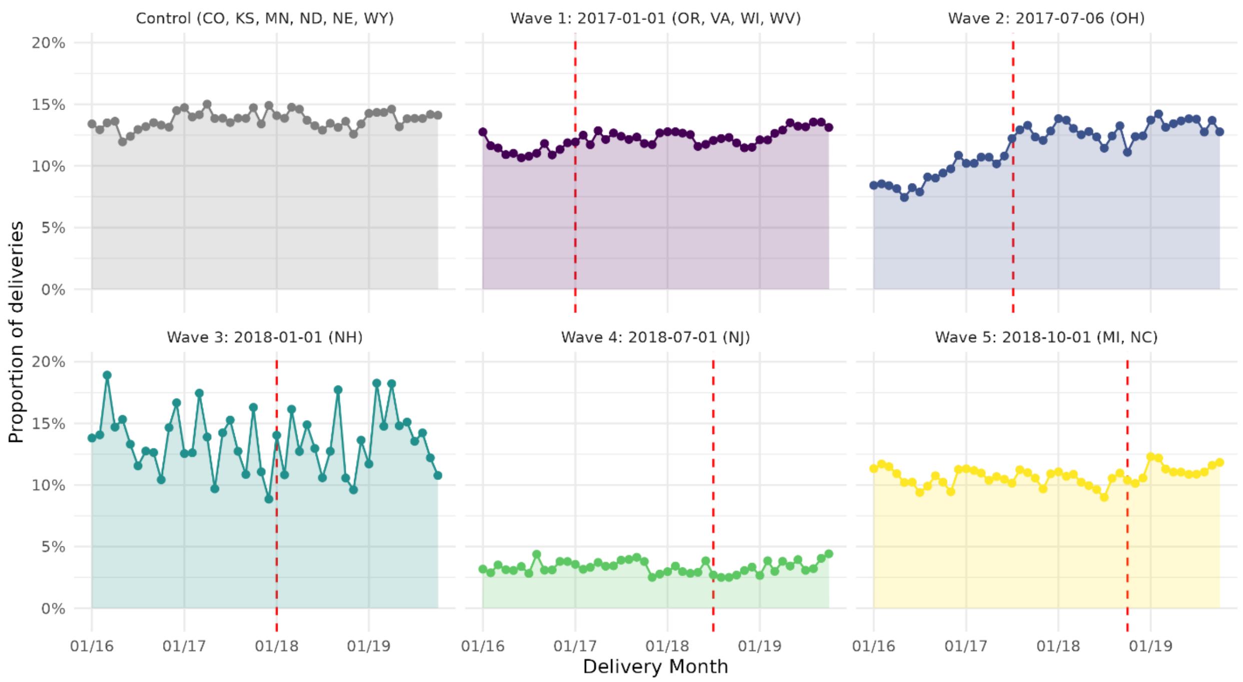

Supplemental eFigure 3. Unadjusted monthly rates of 7-day postpartum sterilization over time stratified by policy implementation wave

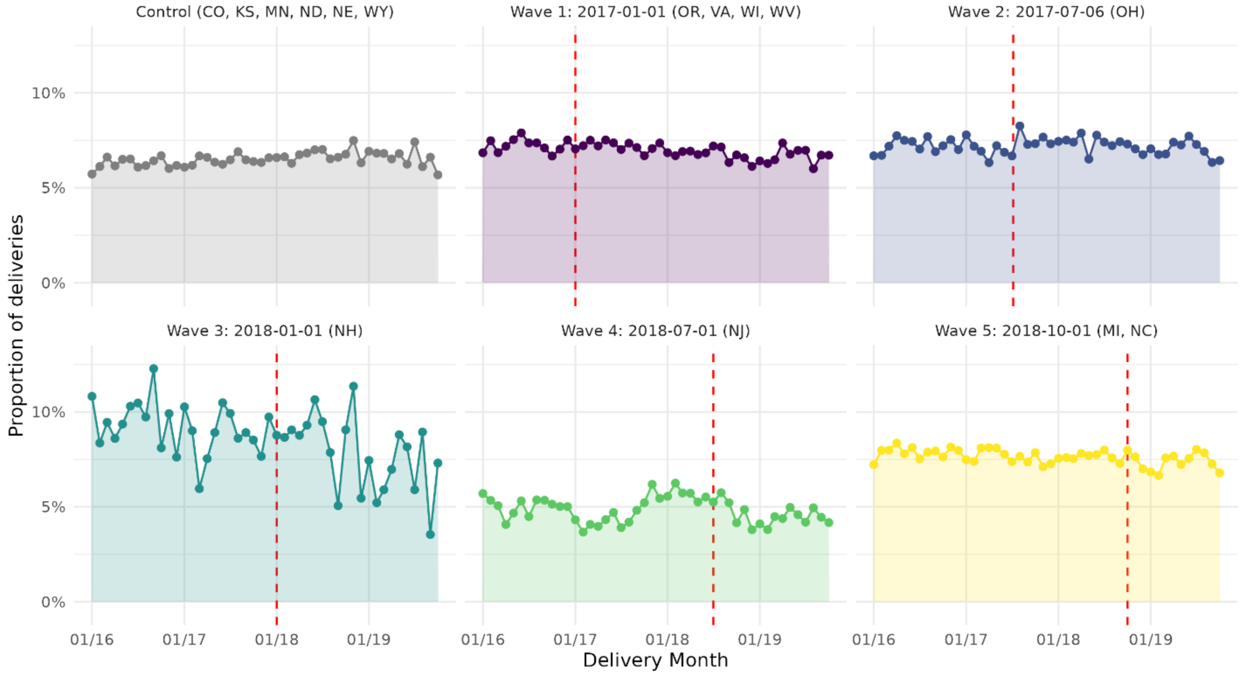

© 2024 Rodriguez MI et al. *JAMA Health Forum.*

Supplemental eFigure 4. Unadjusted monthly rates of 7-day most or moderately effective contraception use over time stratified by policy implementation wave

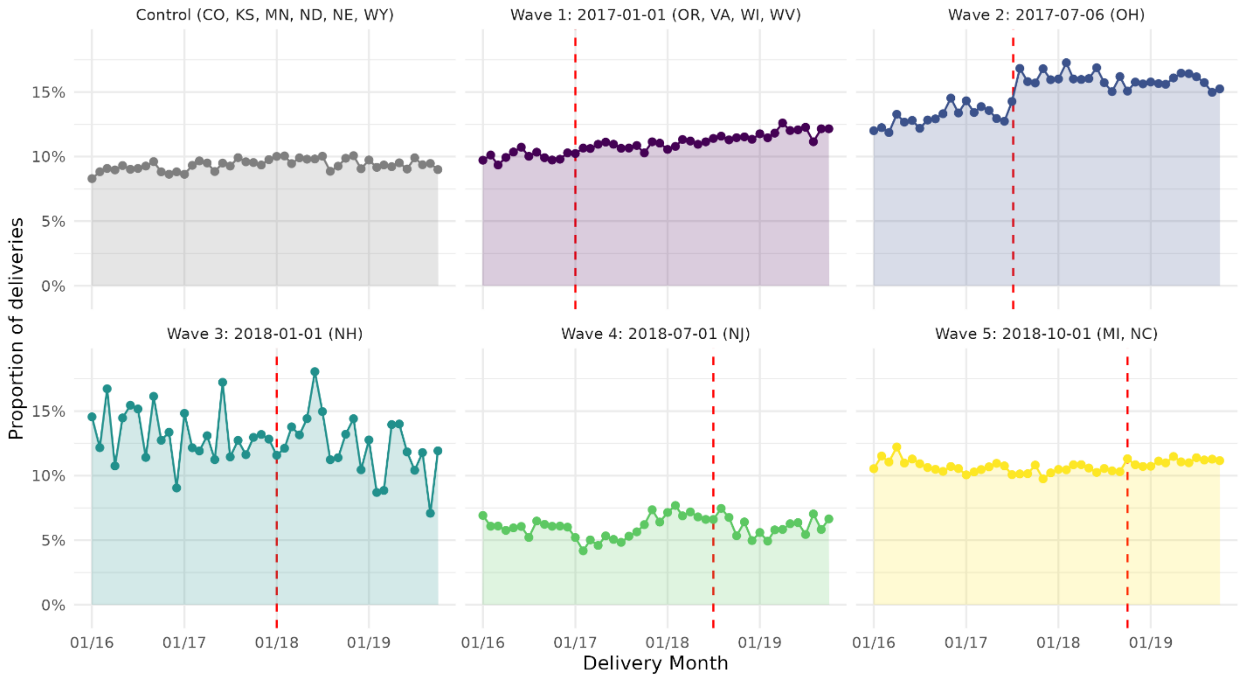

Supplement Section 5. Assessing parallel pre-trends for IPP LARC

One of the assumptions of the staggered difference-in-differences model is that changes over time in each policy wave would follow the same trend as changes over time in the control wave in the absence of treatment. We tested this assumption by estimating time trends for each wave in the pre-policy period using a linear model with interaction terms between policy wave and continuous months since the start of the study. These interaction terms allowed us to test whether those trends significantly differed from the time trend for the control group of states that did not receive the policy. Estimates for the difference in time trends from the control group for each wave and each outcome are presented below:

Supplement eTable 4. Differences in pre-policy trends of IPP LARC by wave

Wave	Estimate	Std. Error	p-value
Wave 1	-0.00005	0.00013	0.665
Wave 2	0.00137	0.00008	<0.001
Wave 3	-0.00059	0.00007	<0.001
Wave 4	-0.00007	0.00007	0.353
Wave 5	-0.000002	0.00008	0.979

© 2024 Rodriguez MI et al. *JAMA Health Forum.*

Supplement Section 6. Sensitivity Analysis for IPP LARC models

To assess the impact of our detrending, we ran a sensitivity analysis on our raw (un-detrended) outcomes. The ATE for the model of raw IPP LARC rates was 1.28 percentage points (95% CI: 0.85, 1.73), slightly higher than the detrended ATE of 0.74 percentage points (95% CI: 0.309, 1.184).

Supplement eFigure 5. Estimated changes each month pre- and post-policy on use of raw IPP LARC rate

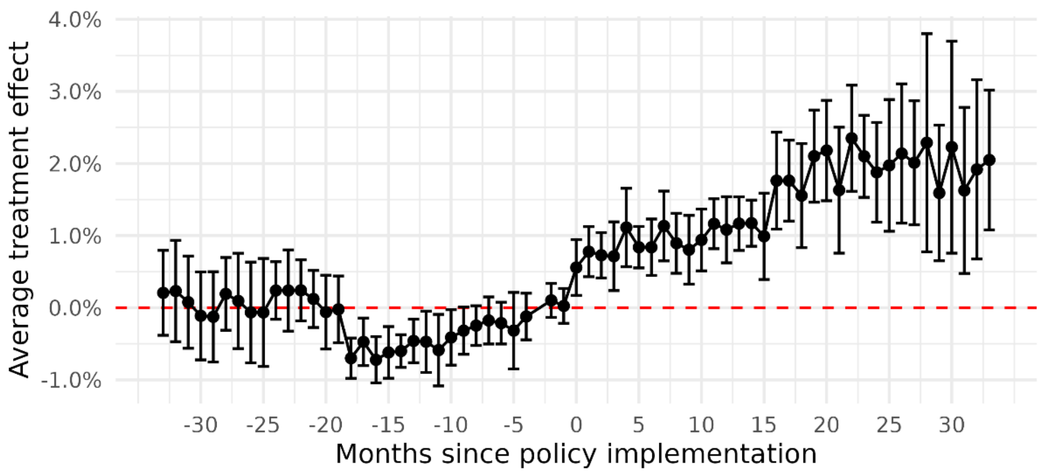

Supplement eFigure 6. Estimated changes associated with the policy for each policy implementation wave on use of raw IPP LARC

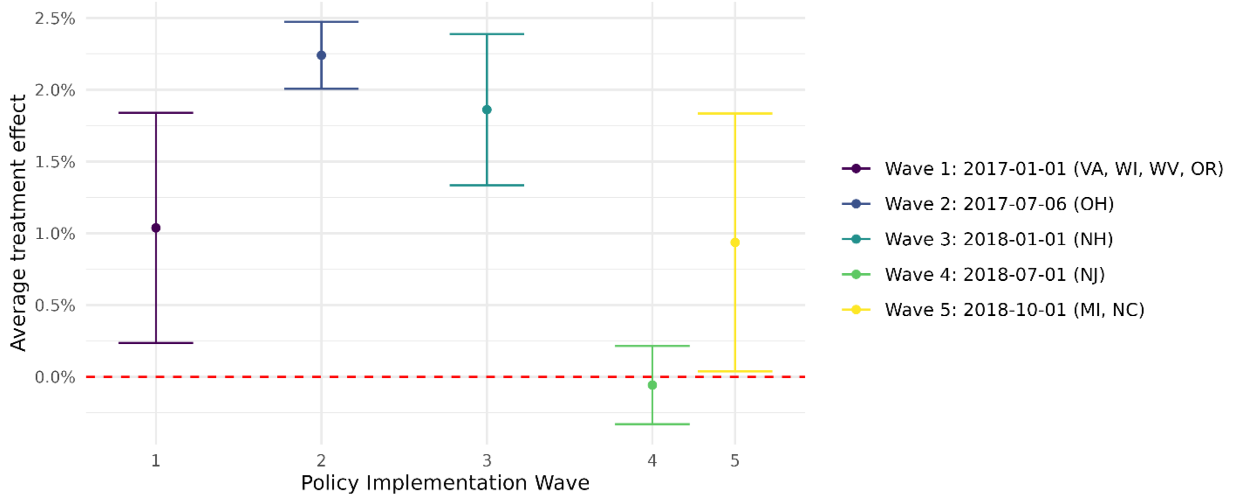

© 2024 Rodriguez MI et al. *JAMA Health Forum.*

Supplement Section 7. Month-stratified and wave-stratified results for secondary outcomes

Supplement eFigure 7. Estimated changes each month pre- and post-policy on use of 60-day postpartum LARC

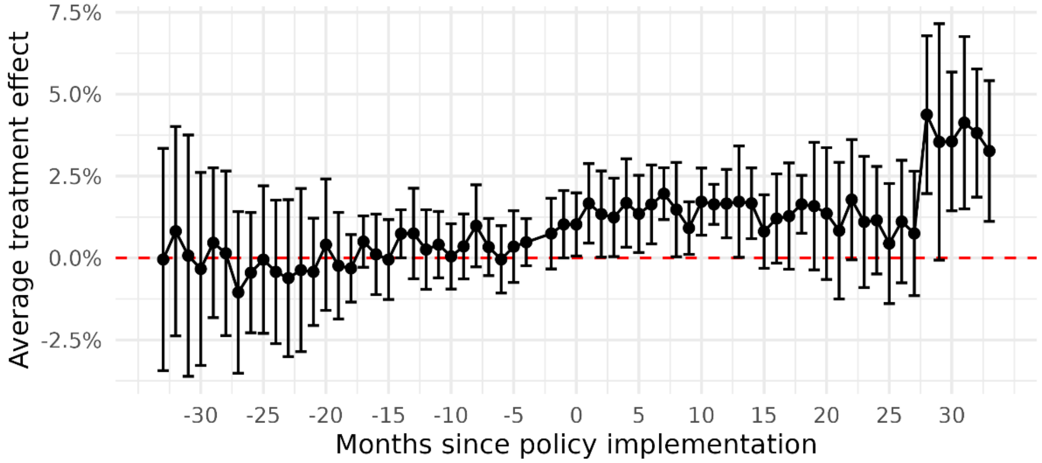

Supplement eFigure 8. Estimated changes associated with the policy for each policy implementation wave on use of 60-day postpartum LARC

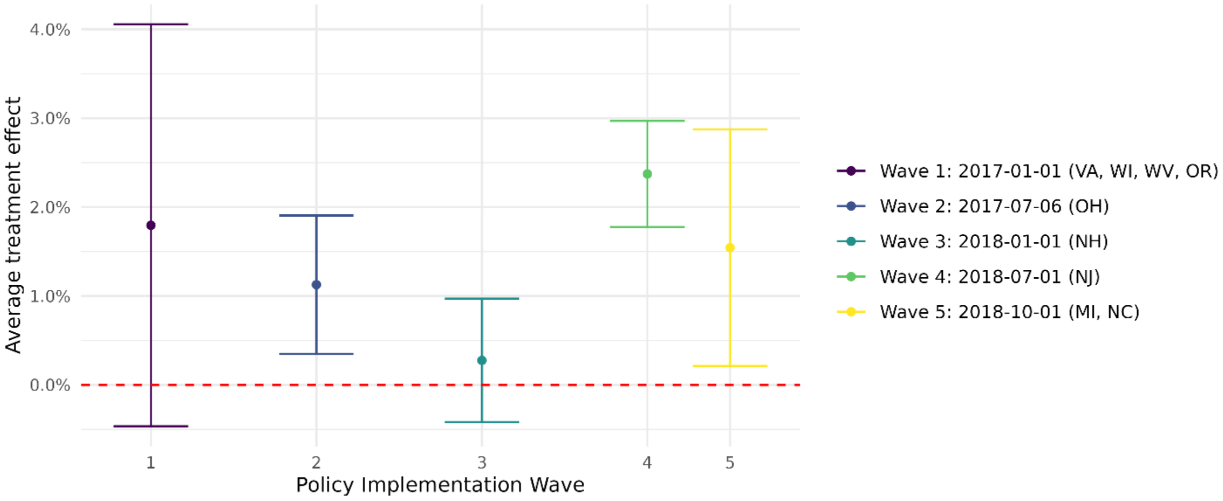

© 2024 Rodriguez MI et al. *JAMA Health Forum.*

Supplement eFigure 9. Estimated changes each month pre- and post-policy on use of 7-day postpartum sterilization

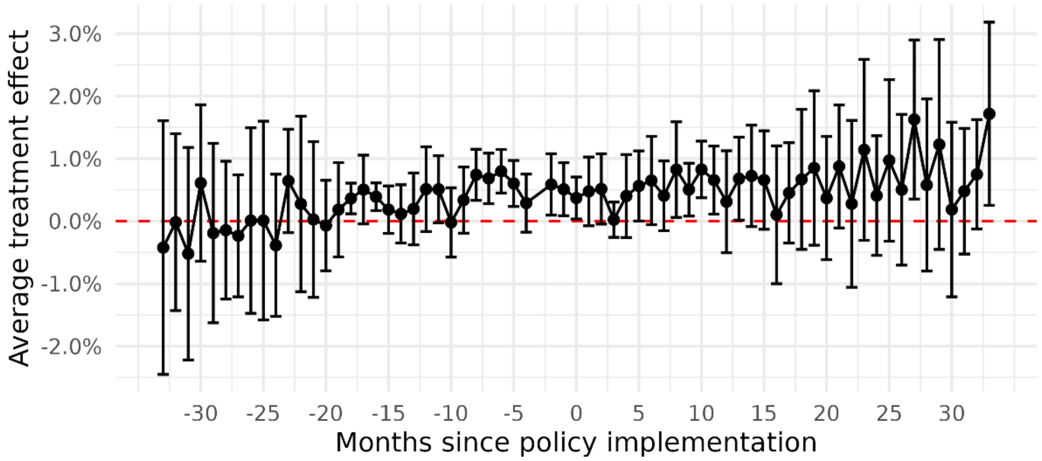

Supplement eFigure 10. Estimated changes associated with the policy for each policy implementation wave on use of 7-day postpartum sterilization

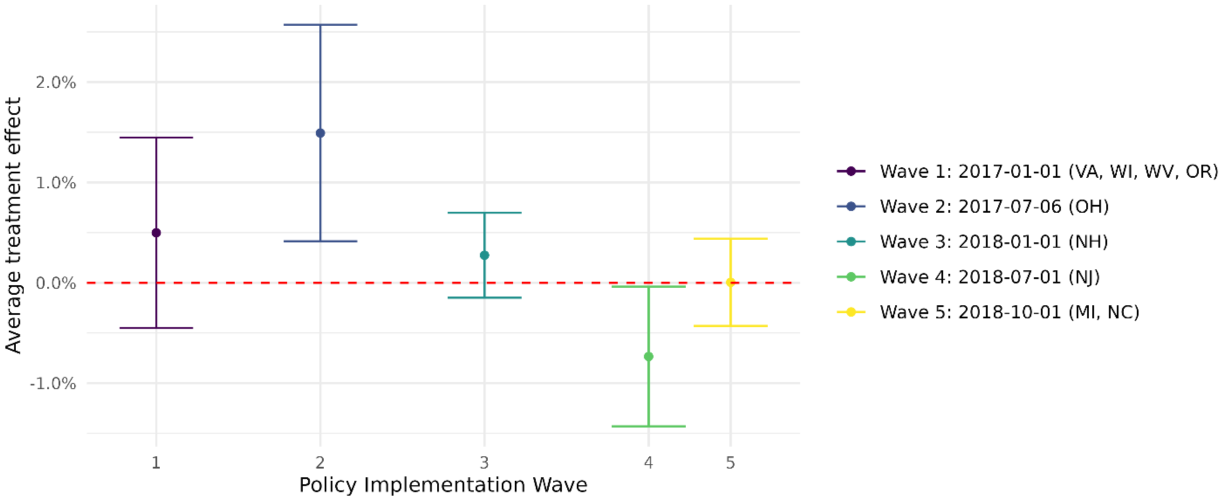

Supplement eFigure 11. Estimated changes each month pre- and post-policy on use of 7-day postpartum most or moderately effective contraception

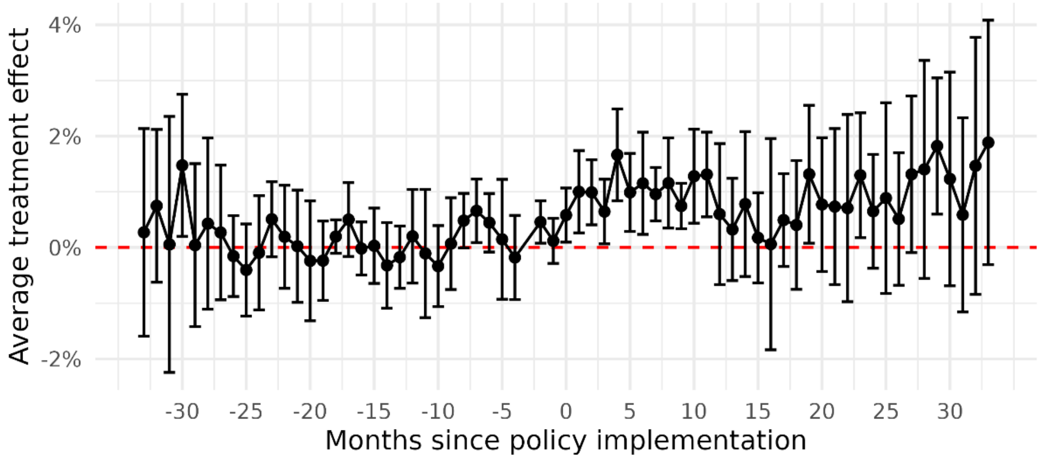

© 2024 Rodriguez MI et al. *JAMA Health Forum.*

Supplement eFigure 12. Estimated changes associated with the policy for each policy implementation wave on use of 7-day postpartum most or moderately effective contraception

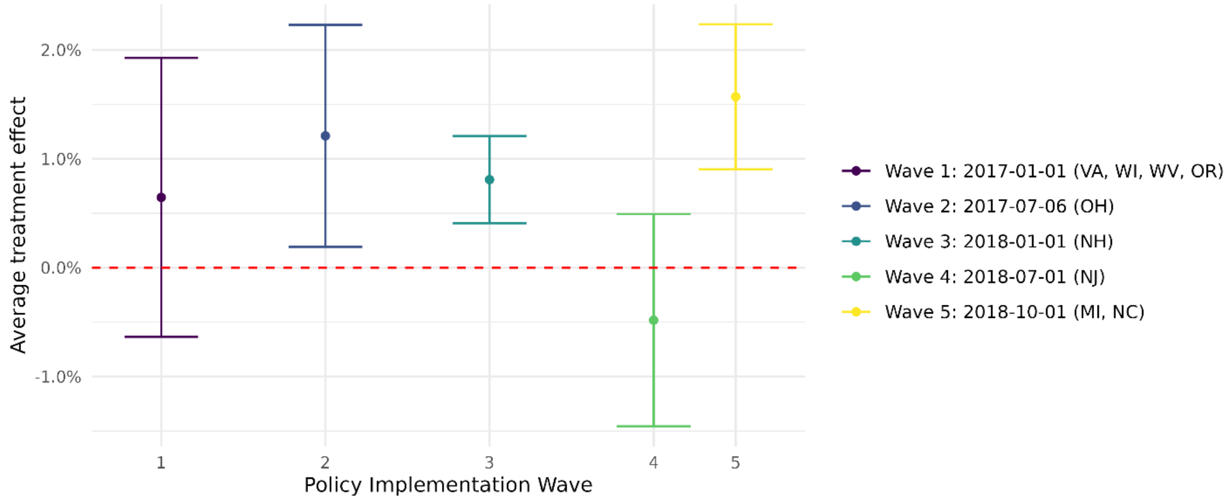

Supplement Section 8. Assessing parallel pre-trends for Secondary Outcomes

Supplement eTable 5. Differences in pre-policy trends of Interval LARC by wave

Wave	Estimate	Std. Error	p-value
Wave 1	-0.00056	0.00035	0.115
Wave 2	0.00132	0.00019	<0.001
Wave 3	-0.00153	0.00012	<0.001
Wave 4	-0.00035	0.00006	<0.001
Wave 5	-0.00004	0.00023	0.081

Supplement eTable 6. Differences in pre-policy trends of 7-day postpartum sterilization by wave

Wave	Estimate	Std. Error	p-value
Wave 1	-0.00028	0.00020	0.161
Wave 2	-0.00022	0.00019	0.250
Wave 3	-0.00057	0.00009	<0.001
Wave 4	- 0.000003	0.00007	0.966
Wave 5	-0.00030	0.00018	0.099

Supplement eTable 7. Differences in pre-policy trends of 7-day postpartum most or moderately effective contraception by wave

Wave	Estimate	Std. Error	p-value
Wave 1	0.00009	0.00037	0.816
Wave 2	0.00081	0.00026	0.002
Wave 3	-0.00077	0.00013	<0.001

© 2024 Rodriguez MI et al. *JAMA Health Forum.*

Wave 4	-0.00004	0.00011	0.708
Wave 5	-0.00053	0.00025	0.033

Supplement Section 9. Sensitivity Analysis for Interval LARC models

To assess the impact of our detrending, we ran a sensitivity analysis on our raw (un-detrended) outcomes. The ATE for the model of raw Interval LARC rates was 1.56 percentage points (95% CI: 0.41, 2.71), almost the same as the detrended ATE of 1.58 percentage points (95% CI: 0.43, 2.73).

Supplement eFigure 13. Estimated changes each month pre- and post-policy on use of raw IPP LARC rate

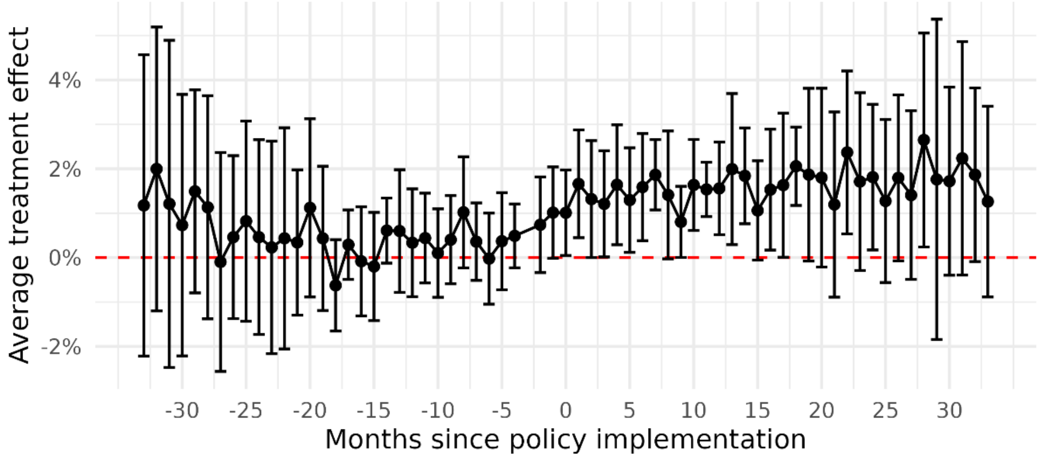

Supplement eFigure 14. Estimated changes associated with the policy for each policy implementation wave on use of raw IPP LARC

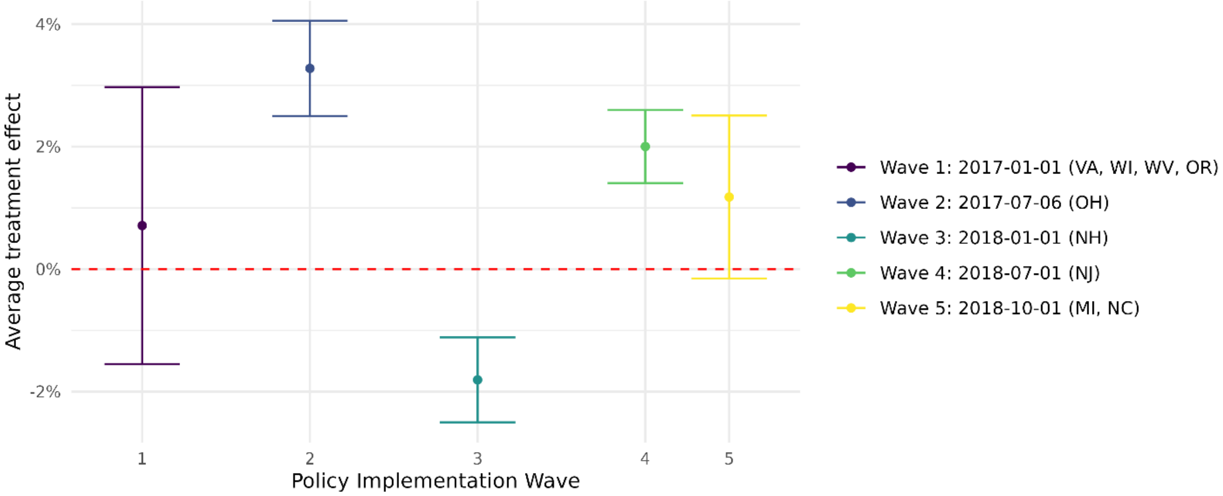

© 2024 Rodriguez MI et al. *JAMA Health Forum.*

Supplement Section 10. Sensitivity Analysis for 7-day postpartum sterilization models

To assess the impact of our detrending, we ran a sensitivity analysis on our raw (un-detrended) outcomes. The ATE for the model of raw 7-day postpartum sterilization rates was 0.18 percentage points (95% CI: -0.44, 0.81), slightly lower than the detrended ATE of 0.59 percentage points (95% CI: -0.03, 1.22).

Supplement eFigure 15. Estimated changes each month pre- and post-policy on use of raw IPP LARC rate

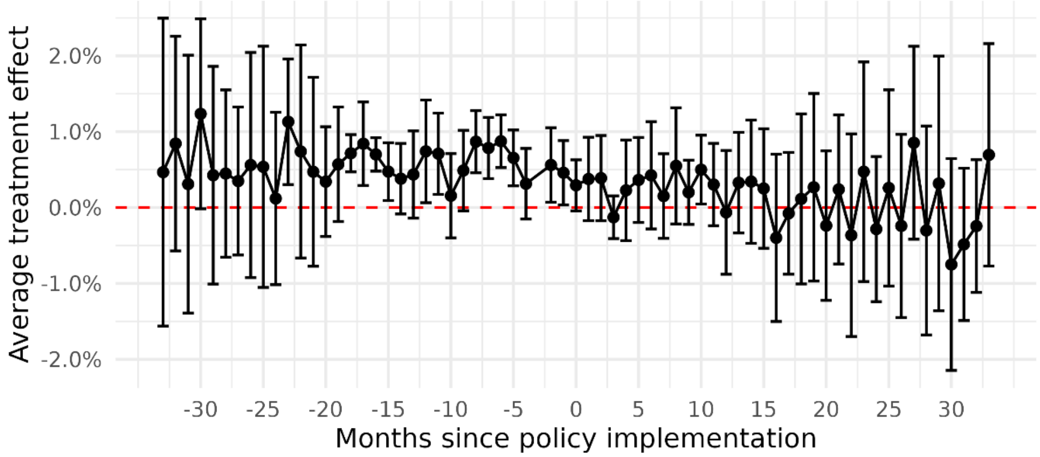

Supplement eFigure 16. Estimated changes associated with the policy for each policy implementation wave on use of raw IPP LARC

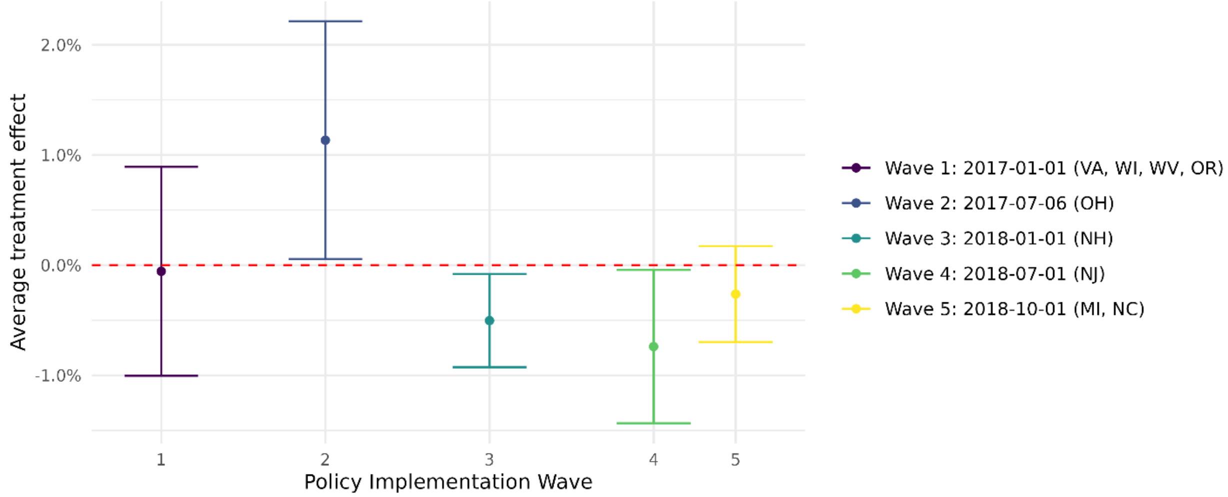

Supplement Section 11. Sensitivity Analysis for 7-day postpartum most or moderately effective contraception models

To assess the impact of our detrending, we ran a sensitivity analysis on our raw (un-detrended) outcomes. The ATE for the model of raw 7-day postpartum most or moderately effective

© 2024 Rodriguez MI et al. *JAMA Health Forum.*

contraception rates was 1.24 percentage points (95% CI: 0.45, 2.03), slightly higher than the detrended ATE of 0.92 percentage points (95% CI: 0.13, 1.71).

Supplement eFigure 17. Estimated changes each month pre- and post-policy on use of raw IPP LARC rate

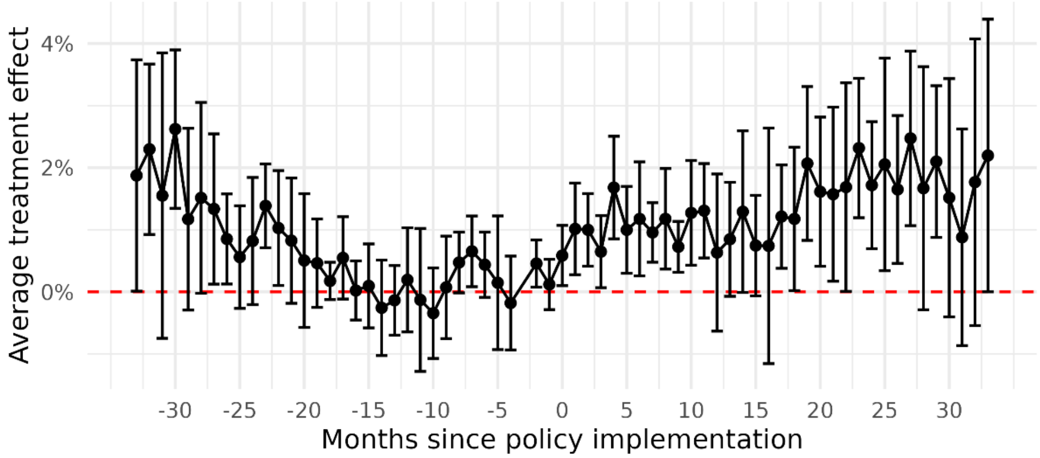

Supplement eFigure 18. Estimated changes associated with the policy for each policy implementation wave on use of raw IPP LARC

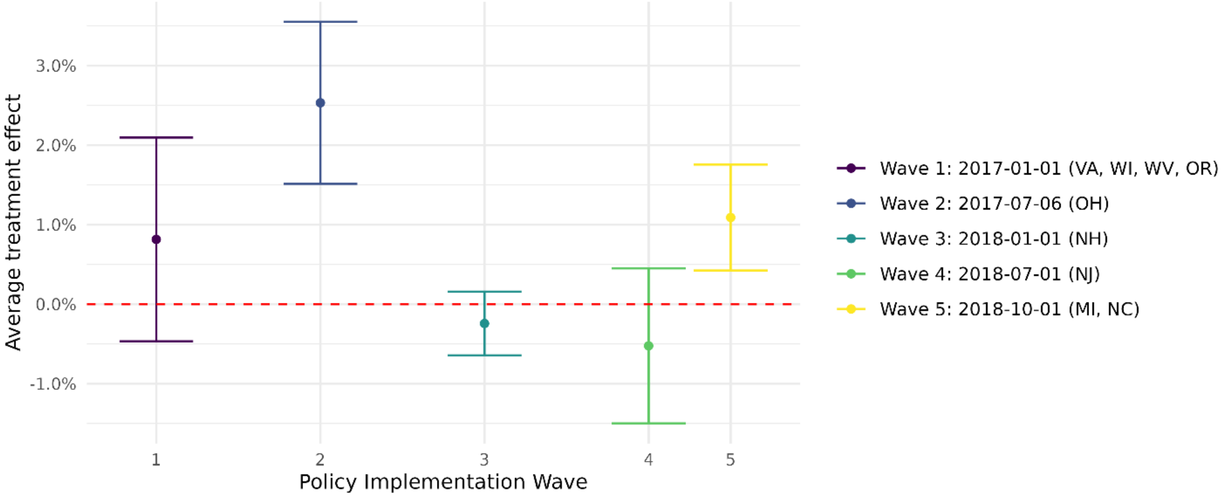

Works Cited

[1] Inventory of State-Level Medicaid Policies, Programs, and Initiatives to Improve Maternity Care and Outcomes. Medicaid and Chip Payment and Access Commission. Accessed September 14, 2023. 7

[2] Center for Medicaid & CHIP Services, Center for Medicare and Medicaid Services, Department of Health and Human Services. CMCS Informational Bulletin: State Medicaid

© 2024 Rodriguez MI et al. *JAMA Health Forum.*

Payment Approaches to Improve Access to Long-Acting Reversable Contraception. 2016. Accessed September 14, 2023. https://www.medicaid.gov/sites/default/files/Federal-Policy-Guidance/Downloads/CIB040816.pdf

[3] Medicaid Reimbursement for Postpartum LARC. American College of Obstetricians and Gynecologists. Accessed September 14, 2023. https://www.acog.org/programs/long-acting-reversible-contraception-larc/activities-initiatives/medicaid-reimbursement-for-postpartum-larc

[4] Ranji, U., Gomez, I., Salganicoff, A. Rosenzweig, C., Kellenberg, R., & Gifford, K. (2022, February 17). Medicaid coverage of family planning benefits: Findings from a 2021 state survey. KFF. https://www.kff.org/report-section/medicaid-coverage-of-family-planning-benefits-findings-from-a-2021-state-survey-report/

[5] Walls, J., Gifford, K., Ranji, U., Salganicoff, A., & Gomez, I. (2016, September 15). Medicaid coverage of family planning benefits: Results from a state survey. KFF. https://www.kff.org/report-section/medicaid-coverage-of-family-planning-benefits-results-from-a-state-survey-reversible-contraception/

[6] Centers for Medicare and Medicaid Services. DQ atlas. Accessed September 14, 2023. https://www.medicaid.gov/dq-atlas/

[7] Abraham S SL. Estimating Dynamic Treatment Effects in Event Studies with Heterogeneous Treatment Effects. Journal of Econometrics. 2021;225.

© 2024 Rodriguez MI et al. *JAMA Health Forum.*
