# Supplementary material for: Medicaid Policy Change and Immediate Postpartum Long-Acting Reversible Contraception
Source: JAMA Health Forum. 2024 Jun 7;5(6):e241359. doi: 10.1001/jamahealthforum.2024.1359 (PMC11161841; doi:10.1001/jamahealthforum.2024.1359)
Supplement: Supplement 3. — Data Sharing Statement [file jamahealthforum-e241359-s003.pdf]

Data Sharing Statement

Rodriguez. Medicaid Policy Change and Immediate Postpartum Long-Acting Reversible Contraception. *JAMA Health Forum*. Published June 07, 2024.
doi:10.1001/jamahealthforum.2024.1359

Data
Data available: No

Additional Information
Explanation for why data not available: This is not allowed under our data use agreement
